# Comparing in vitro human liver models to in vivo human liver using RNA-Seq

**DOI:** 10.1007/s00204-020-02937-6

**Published:** 2020-10-27

**Authors:** Rajinder Gupta, Yannick Schrooders, Duncan Hauser, Marcel van Herwijnen, Wiebke Albrecht, Bas ter Braak, Tim Brecklinghaus, Jose V. Castell, Leroy Elenschneider, Sylvia Escher, Patrick Guye, Jan G. Hengstler, Ahmed Ghallab, Tanja Hansen, Marcel Leist, Richard Maclennan, Wolfgang Moritz, Laia Tolosa, Tine Tricot, Catherine Verfaillie, Paul Walker, Bob van de Water, Jos Kleinjans, Florian Caiment

**Affiliations:** 1 Department of Toxicogenomics, School of Oncology and Developmental Biology (GROW), Maastricht University, Maastricht, The Netherlands; 2 Leibniz Research Centre for Working Environment and Human Factors, Technical University of Dortmund (IfADo), Dortmund, Germany; 3 Division of Drug Discovery and Safety, Leiden Academic Centre for Drug Research, Leiden University, PO Box 9503, 2300 RA Leiden, The Netherlands; 4 Instituto de Investigación Sanitaria La Fe, Experimental Hepatology Unit, Valencia, Spain; 5 Fraunhofer Institute for Toxicology and Experimental Medicine Preclinical Pharmacology and In-Vitro Toxicology, Nikolai-Fuchs-Straße 1, 30625 Hannover, Germany; 6 InSphero AG, Wagistrasse 27, 8952 Schlieren, Switzerland; 7 Department of Forensic Medicine and Toxicology, Faculty of Veterinary Medicine, South Valley University, Qena, 83523 Egypt; 8 In Vitro Toxicology and Biomedicine, Department Inaugurated, Doerenkamp-Zbinden Foundation, University of Konstanz, Konstanz, Germany; 9 Cyprotex Discovery, No 24 Mereside, Alderley Park, Cheshire, SK10 4TG UK; 10 Instituto de Investigación Sanitaria La Fe, Unidad Hepatología Experimental, Valencia, Spain; 11 Stem Cell Institute, Department of Development and Regeneration, KU Leuven, Herestraat 49, 3000 Leuven, Belgium

**Keywords:** In vivo liver, In vitro liver, RNA-seq, CellNet, Non-DEGs, Non-DEGs^DTU−^, Pathway coverage

## Abstract

The liver plays an important role in xenobiotic metabolism and represents a primary target for toxic substances. Many different in vitro cell models have been developed in the past decades. In this study, we used RNA-sequencing (RNA-Seq) to analyze the following human in vitro liver cell models in comparison to human liver tissue: cancer-derived cell lines (HepG2, HepaRG 3D), induced pluripotent stem cell-derived hepatocyte-like cells (iPSC-HLCs), cancerous human liver-derived assays (hPCLiS, human precision cut liver slices), non-cancerous human liver-derived assays (PHH, primary human hepatocytes) and 3D liver microtissues. First, using CellNet, we analyzed whether these liver in vitro cell models were indeed classified as liver, based on their baseline expression profile and gene regulatory networks (GRN). More comprehensive analyses using non-differentially expressed genes (non-DEGs) and differential transcript usage (DTU) were applied to assess the coverage for important liver pathways. Through different analyses, we noticed that 3D liver microtissues exhibited a high similarity with in vivo liver, in terms of CellNet (C/T score: 0.98), non-DEGs (10,363) and pathway coverage (highest for 19 out of 20 liver specific pathways shown) at the beginning of the incubation period (0 h) followed by a decrease during long-term incubation for 168 and 336 h. PHH also showed a high degree of similarity with human liver tissue and allowed stable conditions for a short-term cultivation period of 24 h. Using the same metrics, HepG2 cells illustrated the lowest similarity (C/T: 0.51, non-DEGs: 5623, and pathways coverage: least for 7 out of 20) with human liver tissue. The HepG2 are widely used in hepatotoxicity studies, however, due to their lower similarity, they should be used with caution. HepaRG models, iPSC-HLCs, and hPCLiS ranged clearly behind microtissues and PHH but showed higher similarity to human liver tissue than HepG2 cells. In conclusion, this study offers a resource of RNA-Seq data of several biological replicates of human liver cell models in vitro compared to human liver tissue.

**Electronic supplementary material:**

The online version of this article (10.1007/s00204-020-02937-6) contains supplementary material, which is available to authorized users.

## Introduction

The liver plays a central role in metabolizing exogenous substances. After oral uptake xenobiotics pass through the digestive tract and enter the liver via the portal vein, where metabolism by phase I and II enzymes take place (Cribb et al. 2005). Xenobiotics or their metabolites may damage the liver with fatal consequences for the individual (Moeller et al. 2012). Therefore, it is important to identify compounds that cause hepatotoxic effects to avoid exposure to humans.

While the use of animal models has proven to be of great importance in biological research (Dey et al. 2010; Ericsson et al. 2013; Hau 2008; Simmons 2008), it remains challenging to translate the results to humans. Many drugs that showed great promise in animal testing failed safety assessment in clinical trials, e.g. emicizumab, zydelig, JCAR014, JCAR015, and Ad-RTS-hIL-12. To overcome these limitations, human cell models have emerged as a viable alternative for efficacy, safety, and toxicity testing (DelRaso 1993; Godoy et al. 2013). These in vitro models do not just eliminate the species-specific variations but also have other advantages such as the requirement of only small amounts of the substance, relatively short testing periods, and the technically easy possibility to study mechanisms of toxicity, enzyme kinetics, and concentration–response relationships (DelRaso 1993; LeCluyse et al. 1996). Limitations using in vitro cell models are that differences between cells in vitro and in vivo may exist; moreover, relatively complex techniques are required to extrapolate from test compound concentrations in the culture medium in vitro to blood concentrations or doses in vivo (Albrecht et al. 2019; Sachinidis et al. 2019).

Several human liver cell models have been developed with an aim to resemble the in vivo situation as closely as possible (Gebhardt et al. 2003). HepaRG cells may be used for xenobiotic metabolism, toxicity studies, cytochrome P450 induction studies, and for analyzing genotoxic compounds (Guillouzo et al. 2007; Kanebratt and Andersson 2008). Primary human hepatocytes are still considered to represent a gold standard for hepatic biotransformation studies (Godoy et al. 2018; Gu et al. 2018), whereas HepG2 cells have been reported to represent a useful tool to study the regulation of drug-metabolizing enzymes (Wilkening et al. 2003). In a review of different in vitro liver cell models, the advantages and disadvantages of the in vitro liver cell models have also been discussed (Soldatow et al. 2013). Though informative, these studies only give a superficial comparison as they are based on selected processes and components, whereas next-generation sequencing (NGS) technologies can be used to obtain an unbiased, holistic view.

The evolution of NGS over the years has revolutionized genomics and transcriptomics research (Van Dijk et al. 2014) making it affordable, fast, and precise. With NGS-based RNA-sequencing (RNA-Seq), it has now become possible to both identify and quantify RNA transcripts (Chu and Corey 2012), even in the absence of any prior genomic knowledge (Van Dijk et al. 2014; Wang et al. 2009). Quantification of the transcript level, known as gene expression, can be analyzed in many different ways (Bae et al. 2016; Chen et al. 2011; DelRaso 1993; Kvam et al. 2012) depending on the type of biological questions that need to be addressed. RNA-Seq provides the exhaustive expression profile of all genes expressed in the cell and is not limited to a set of genes widely studied.

In this study, we compared healthy human liver tissue, further referred to as “in vivo liver” with in vitro liver cell lines often used in toxicology studies. For the bioinformatics analysis, we used CellNet (Cahan et al. 2014a) which is a network biology-based computational platform to assess RNA-Seq expression data. In CellNet, consensus expression profiles of specific cells or tissue types were generated. For the ease of use, the authors have created transcriptome indices and annotation files of some cells/tissues by congregating publicly available RNA-Seq data for humans. We used these human indices and annotation files for comparing the liver in vitro cell models. Comparing the consensus expression data with the test cell models objectifies their similarity with different cells/tissues. CellNet also creates gene regulatory networks (GRN) that are derived from the expression profile. GRN is a network of genes that interact with each other to control-specific cell functions (Li and Davidson 2009). GRNs can also be used to analyze similarities as they are specific for development, differentiation, and response to environmental cues (Godoy et al. 2015).

To study each component (genes and/or transcripts) individually with equal weight, we also analyzed non-differentially expressed genes (non-DEGs). Usually, differentially expressed genes (DEGs) between samples are analyzed to describe the differences between cell types, exposures, time points, or other influences (Kvam et al. 2012). Here, also the non-DEGs were analyzed to focus on the similarities between in vitro liver cell models and in vivo liver. The higher the number of non-DEGs the higher the similarity an in vitro model and the liver.

Gene expression levels from RNA-Seq data are usually obtained by summing the reads attributed to all transcript (or isoforms) variants for each given gene so that the change in the amount of expression of individual isoforms is not apparent. A previous consensus has been that the majority of genes are regulated through their mRNA levels but NGS has shown that also the selection of individual spliced variants may change, while the sum of all isoforms remains unchanged. Moreover, the ENCODE project revealed through NGS that close to 95% of human multi-exon genes undergo alternative splicing (Carninci 2009) to form the gene transcripts. Gene transcripts are mRNAs that have different transcription start sites (TSSs), protein coding DNA sequences (CDSs), and/or untranslated regions (UTRs) but all are expressed from the same locus. Ensembl (Aken et al. 2016) provides an extensive list of transcript types broadly categorized as protein coding, nonsense mediated decay, non-stop decay, and long as well as small non-coding RNA (https://www.ensembl.org/info/genome/genebuild/biotypes.html). By RNA-Seq, quantified information of different transcripts of a gene can be obtained (Van Dijk et al. 2014) and changes in the fraction of each transcript, known as differential transcript usage (DTU), can be studied to provide insights. These differences in the ratio of the expression of the transcripts can potentially alter the gene function and the mRNA regulation, stability, and localization (Matoulkova et al. 2012; Mayr 2016).

To our knowledge, previous studies have compared genome-wide expression only of individual cell models, such as e.g. PHH and iPSC derived human hepatocyte-like cells (Godoy et al. 2015, 2018); however, a systematic comparison of the most frequently applied in vitro liver cell models to human liver tissue has not yet been performed. Here, we studied different in vitro liver cell models at baseline conditions, i.e. without any compound exposure: liver models derived from cancer cells (HepG2, HepaRG 3D), iPSC (induced pluripotent stem cells)-derived hepatocyte-like cells, cancerous human liver-derived (hPCLiS, human precision cut liver slices), and non-cancerous human liver-derived cultivated primary human hepatocytes (PHH) and 3D liver microtissues. These cell models were compared to healthy in vivo liver assessed by NGS data.

## Materials and methods

An overview of the analyzed samples, human liver tissue specimens in vivo and in vitro liver cell models is given in Table 1, detailed information on samples and protocols is available in Suppl. methods 1a-g, and details on samples selected after each filtration step are provided in Suppl. Table 1. For PHH, hPCLiS, and 3D liver microtissues expression data for multiple time points are available.

**Table 1:** Overview of in vitro liver cell models used in this study

Cell line	Cultivation period (in h)	No of replicates (biological/technical)			Protocol and other details
		Total	Sequencing depth filtration	After bootstrapping (replicates most similar to in vivo healthy liver)	
In vivo liver	NA	27	24^c^	24	Suppl. methods 1(a)
PHH	0^a^	6	6	3	Suppl. methods 1(b)
	24	6	6	3	
iPSC-HLC	480	6^b^	3	3	Suppl. methods 1(c)
3D liver microtissues	0^a^	3	2	2	Suppl. methods 1(d)
	168	3	3	3	
	336	3	3	3	
HepG2	0^a^	7	7	3	Suppl. methods 1(e)
HepaRG 3D	0^a^	4	3	3	Suppl. methods 1(f)
hPCLiS	0^a^	4	4	3	Suppl. methods 1(g)
	24	4	4	3	

^a^Timepoint 0 h is time post-seeding

^b^Donor 1 (SBAD2) → 4 replicates; Donor 2 (SBAD3) → 2 replicates

^c^3 samples from infants or children were removed

### RNA sequencing

All samples from the in vitro liver cell models were analyzed by a standardized working pipeline that included the immediate transfer of cells and tissues into TRIzol™ after the cultivation periods as indicated in Table 1. RNA was extracted from these cell samples with a Qiagen miRNeasy Mini Kit (Cat # 217004). Additionally, DNase digest was performed with a Qiagen RNase-Free DNase Set (Cat # 79254) to remove unwanted DNA. RNA quantity and quality were assessed by Qubit™ RNA HS Assay Kit (Cat # Q32855) and Agilent RNA 6000 Nano Kit (Cat #5067-1511) respectively and prepared for sequencing with the Lexogen SENSE mRNA-Seq Library Prep Kit V2 (Cat # 001.96). After library preparation was completed, the quality of the libraries was checked on an Agilent 2200 TapeStation using an Agilent High Sensitivity D5000 ScreenTape (Cat # 5067-5592) and library concentration was determined by Qubit™ dsDNA HS Assay Kit (Cat # Q32854) before proceeding to sequencing. While the healthy liver tissue samples were sequenced (paired-end, 150 bp) on an Illumina NovaSeq 6000® using a single S2 flowcell, the in vitro cell models were sequenced (paired-end, 100 bp) on Illumina HiSeq2000®.

### Data pre-processing

The quality of the RNA-Seq raw data (fastq files) was analyzed using the Fastqc (version 0.10.1) (Langmead and Salzberg 2012) and after considering the quality of the sequences, tails of the sequences were trimmed of the bad quality of the sequences (twelve nucleotides) using Trimmomatic (version 0.33) (Bolger et al. 2014). The sequences were mapped onto the Ensembl (Aken et al. 2016) human genome (version 84) using Bowtie2, (version 2.2.6) (Langmead and Salzberg 2012), and gene and isoform (transcript) expression were calculated using RSEM (version 1.2.28) (Li and Dewey 2011). Using the sorted genome bam files from RSEM, annotation of the mapped reads was assessed by applying ALFA (Bahin et al. 2019).

The gene read counts, isoform read counts and isoform percentage from all in vivo and in vitro samples were taken. Gene read counts were used for finding the non-DEGs, then isoform count and percentage were used to analyze DTU. Calculation of non-DEGs and DTU is done for each cell model and all time-points, individually.

### CellNet analyses

The fastq files were used for CellNet analysis. All the subsequent steps were performed locally as explained in the CellNet protocol paper (Radley et al. 2017). We used the ‘Human Jun_20_2017’ cnProc from the CellNet for analyses. Two types of analyses were performed: comparing the consensus expression profile and GRN status. The consensus expression profile per cell or tissue type is generated from publicly available RNA-Seq data and classification scores for the test samples are obtained. GRN created from the consensus expression profile give the GRN status when samples are compared against them. These are calculated by first computing the raw GRN status as the mean z-score of all genes in a C/T (cell/tissue) GRN, weighted by their importance to the associated C/T classifier. The raw GRN status is then normalized to the mean raw GRN status of the training data samples of the given C/T (Cahan et al. 2014b).

### Bootstrapping

A different number of replicates (technical or biological) were present for all cell models. To eliminate this possible source of bias, we selected three replicates from each cell model which presented the highest similarity with the healthy in vivo liver (Table 1, Suppl. Table 1) based on the number of non-DEGs. In the case of 3D liver microtissues, only two replicates were taken instead of three, because the third replicate had very low coverage and was discarded at the sequencing depth filtration. These selected replicates were then used to calculate non-DEGs, DEGs, DTU, and other further analyses.

### Non-DEGs

The data were normalized by defining in vivo liver as one dataset and each in vitro liver model for each time point as an individual dataset (best three replicates were taken as explained above). Then each in vitro dataset was compared individually to the in vivo dataset for calculating the non-DEGs using the ‘DESeq’ function from DESeq2 R-package (Love et al. 2014). The list of non-DEGs is obtained by filtering the results for q value (padj) > 0.05 and basemean > 10. These non-DEGs were mapped onto KEGG pathways (Kanehisa et al. 2004) using Pathview (Luo and Brouwer 2013), and in-house developed scripts were used to calculate the pathway coverage.

### Differential transcript usage (DTU)

The change in the proportion of the transcripts expressed for a gene represents differential transcript usage. Isoform counts and percentages were calculated using RSEM. The isoform counts were normalized using DESeq2 as explained for gene reads for the selected replicates for each cell model. Considering the number of transcripts assessed, multiple filtering steps were applied to remove the low expressed transcripts (or noise), and transcripts expressed at a similar level from the control (in vivo) and test (in vitro) samples.

1. Low expression/noise:
  Isoforms that were expressed less than one in a million reads in one dataset (test or control) were removed. These isoforms were removed, because their expression level was not sufficient to be considered above the noise at this sequencing depth. This filtration step was performed on isoform counts.
2. Similar expression:
  Isoforms that differ less than equal to 10% between the average percentage of in vivo and in vitro samples were removed, because we were interested in looking for the isoforms having sufficient differential usage. This filtration step was performed on isoform percentages.
3. No expression in some samples:
  The isoforms that were not detected in more than 20% of the samples in any one of the datasets (in vivo or in vitro) were discarded, as this would reduce the confidence in the samples that showed expression for those isoforms. If the number of samples for test or control were less than five, we imposed that the transcripts were detected in all samples. This filtration step was performed on isoform percentages.

Isoforms deleted from the count dataset were removed from the percentages dataset and the ones deleted from the percentages were removed from the counts.

After the filtration steps, the genes left with only one isoform were removed from both datasets (counts and percentages). The variance was calculated between the test and control samples for all the remaining transcripts using ANOVA in R. Isoform percentages were used to find the variance because percentages are linearly distributed (contrary to the RNA-Seq read count). It was filtered on *p* value < 0.01 as the calculation at the isoform level has a higher error rate.

The highest expressed isoform was identified (highest percentage) in the control samples for each gene and was compared with its expression profile in test samples. The genes with different expression profiles (DTU) were removed from the non-DEGs and were named as non-DEGs^DTU−^. The list of non-DEGs^DTU−^ was mapped onto the KEGG pathways and pathway coverage was recalculated.

## Results

RNA from totally of 27 liver tissue specimens from donors without liver diseases, further named “healthy in vivo liver” and 46 samples from cultivated hepatocytes, cell lines, liver slices or iPSC-derived hepatocyte-like cells, so-called in vitro cell models, were sequenced on the Illumina NovaSeq (PE, 150 bp) and the Illumina HiSeq 2000 (PE, 100 bp), respectively. After removing the samples from children or infants for healthy in vivo liver specimens and filtering for sequencing depth 24 in vivo cell models, 38 in vitro samples remained for further analysis (Suppl. Table 1). Since healthy human liver represents a very valuable resource, we generated sequences at very high depth (1.63*10^8^) for community usage. However, to avoid coverage bias in our analysis with the in vitro samples (sequenced at a depth of 33.65*10^6^), only the first 30 million reads of the fastq files obtained from the in vivo samples were used. We compared the full coverage and part of the data and found that the whole sample and sub-selection had similar distribution (Suppl. Figure 1).

**Fig. 1:**
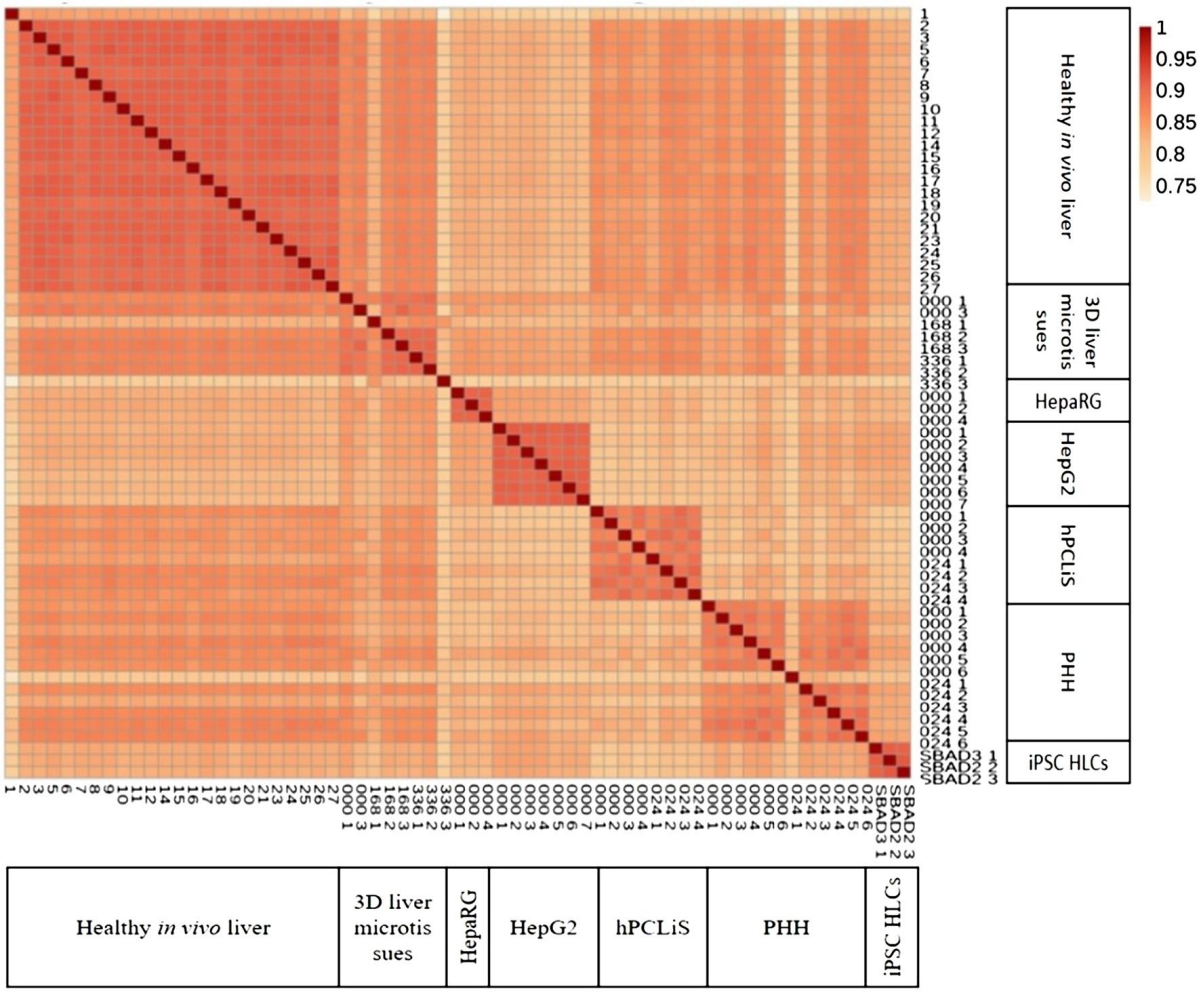
Spearman’s correlation plot. The Spearman’s correlation plot for normalized read counts of all in vivo and in vitro samples taken after first sample filtration. For healthy in vivo liver, the replicate numbers are given. For all in vitro cell models, cultivation periods (000/024/168/336 h) and replicate numbers are indicated except iPSC-HLC. In the case of iPSC-HLC, the donor id (SABD2/3) and replicate number are given. The color bar indicates the Spearman correlation coefficient of each pairwise correlation

All samples taken after initial filtration passed the ‘per base sequence quality’ metric of the Fastqc. The annotation of reads was assessed using ALFA. Median protein coding reads were 47.02%, 52.3%, 45.52%, 40.9%, 40.87%, 35.15%, and 47.16% for healthy liver tissue*,* 3D liver microtissues, HepaRG, HepG2, hPCLiS, PHH, and iPSC-HLC, respectively (Suppl. Figure 2a and b). The samples that had lower protein coding and 3′UTR reads showed an increase in the intergenic and 5′UTR reads. Overall, the samples had similar distributions across different regions. Furthermore, the global similarity of the cell models was evaluated using pairwise Spearman’s correlation for normalized read counts (Fig. 1). The median (and standard deviation) of all pairwise correlation coefficients of the healthy liver tissue specimens with the samples from 3D liver microtissues, HepaRG, HepG2, hPCLiS, PHH, and iPSC-HLC, were 0.87 (0.033), 0.83 (0.008), 0.82 (0.013), 0.86 (0.014), 0.86 (0.014), and 0.83 (0.01), respectively. The variation coefficients of 3D liver microtissues, HepaRG, HepG2, hPCLiS, PHH, and iPSC-HLC, were 3.87, 0.99, 1.57, 1.65, 1.59, and 1.22, respectively. Inter-replicate variation was observed predominantly in 3D liver microtissues (166_1 and 336_3) and PHH (024_1) cell models. It should be considered that interindividual variability contributes to the cell models obtained from different donors (healthy liver tissue specimens, 3D liver microtissues, hPCLiS, PHH), in contrast to the cell line derived cell models (iPSC-HLC, HepaRG, HepG2).

### CellNet cell/tissue classification scores of RNA-Seq expression profiles

**Fig. 2:**
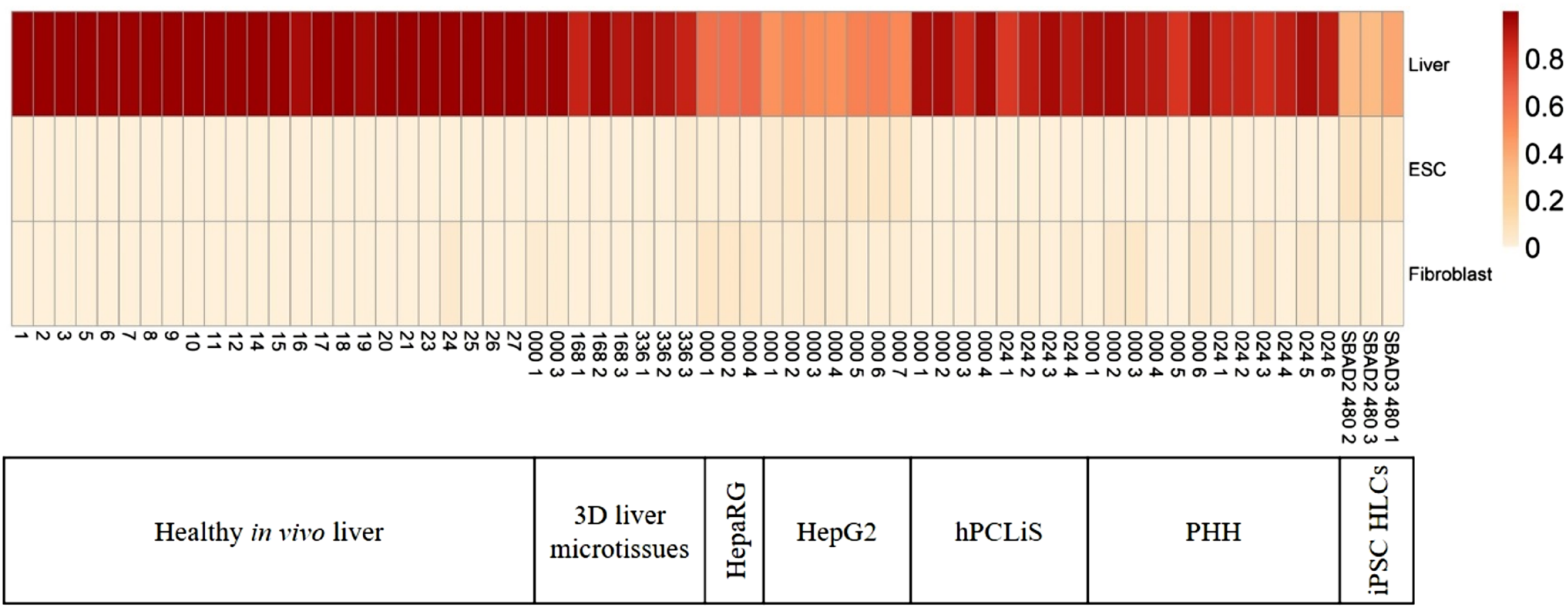
CellNet C/T classification score. The classification score for in vivo and in vitro samples compared to the liver, embryonic stem cell (ESC), and fibroblast data of CellNet, represented as a heat map. For healthy in vivo liver, the replicate numbers are indicated. For all in vitro cell models, time points (000/024/168/336 h) and replicate numbers are given except iPSC-HLC. In the case of iPSC-HLC, the samples are labeled as donor id (SABD2/3) and replicate number. The color bar represents the classification score as calculated by CellNet

**Fig. 3:**
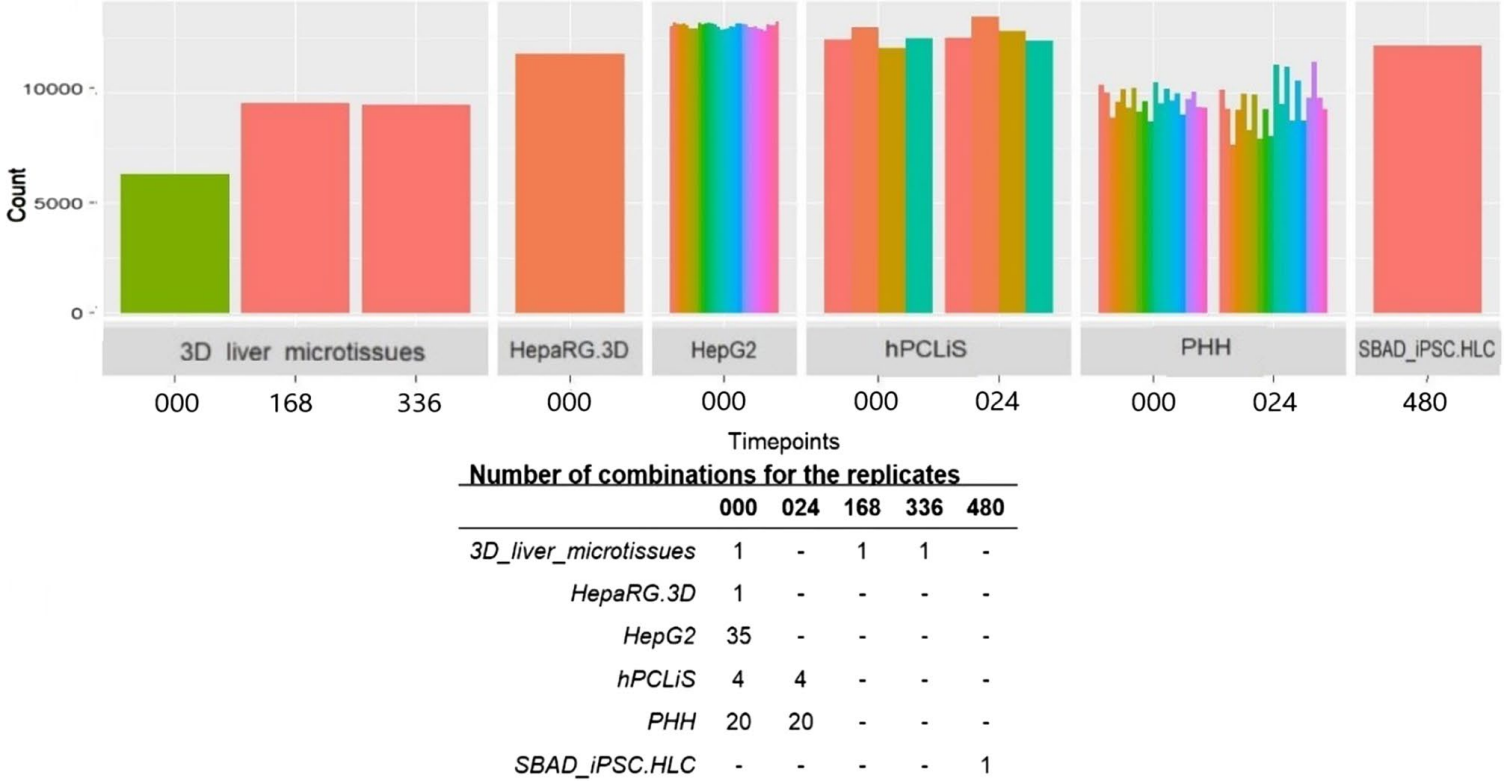
Number of DEGs from combination of replicates during bootstrapping. There were variable number of replicates for the cell models. This may result in incomparable statistical analyses of the cell models. Therefore, to address this concern, a bootstrap strategy was applied to select the replicates that had the least number of DEGs when compared to in vivo liver. Different colors of the bar graph represent various combinations of the replicates

Since the quality and global distributions of the samples were comparable, we next assessed their transcriptome expression to consensus profiles of different cells and tissues. Using CellNet on the expression data of our liver in vivo and in vitro samples, we calculated classification scores (Fig. 2; Suppl. Table 2). CellNet classified all cell models as liver. We noticed that the iPSC-HLCs present the lowest CellNet classification score for the human liver and they still share some resemblance with embryonic stem cells (ESC). Among all the cell models, HepaRG 3D had the highest classification score for fibroblasts. The cancer cell models (HepG2 and HepaRG 3D) also exhibited low classification scores as compared to non-cancerous liver-derived cell models, whereby the classification scores of HepaRG 3D were slightly higher compared to HepG2 cells but still much lower than the values for 3D liver microtissues, hPCLiS, and iPSC-HLC. The human liver-derived models (3D liver microtissues, hPCLiS, and iPSC-HLC) did not show major differences among each other based on the CellNet classification score. Furthermore, at the level of GRNs (status score) (Suppl. Figure 3), similar results were obtained as for the consensus expression comparison (classification score). CellNet results can be used to find the extent of similarity and dissimilarity for the cell models but other approaches should be used to identify the differences at gene and transcript level. In this study, we explored non-differentially expressed genes (non-DEGs) and differential transcript usage (DTU) to provide comprehensive comparisons between the cell models. However, to remove the bias caused by the different number of replicates from each cell model, we performed bootstrap analyses to guarantee that an identical number of replicates from each cell model was used (Fig. 3; Suppl. Table 1). The cell models had differing number of replicates, hence the number of combinations of replicates, taken three at a time, also varied across cell models. The number of DEGs for 3D liver microtissues were 6315 (0 h), 9552 (168 h), and 9478 (336 h), for iPSC-HLCs, it was 12,155 (480 h), and 11,790 for HepaRG 3D. For these cell models, only one combination of replicates per time point was obtained. For the remaining cell models, where the number of replicates were more than three after the initial filtration for quality, the average number of DEGs for PHH were ~ 9684 (0 h) and ~ 9508 (24 h), for hPCLiS the mean was ~ 12,499 (0 h) and ~ 12,815 (24 h), and for HepG2 it was 13,070 (0 h). From these, the best three replicates were selected based on the number of non-DEGs, except for 3D liver microtissues 0 h, because one of the replicate was discarded for low coverage.

### In vivo versus in vitro, using non-differentially expressed genes

**Fig. 4:**
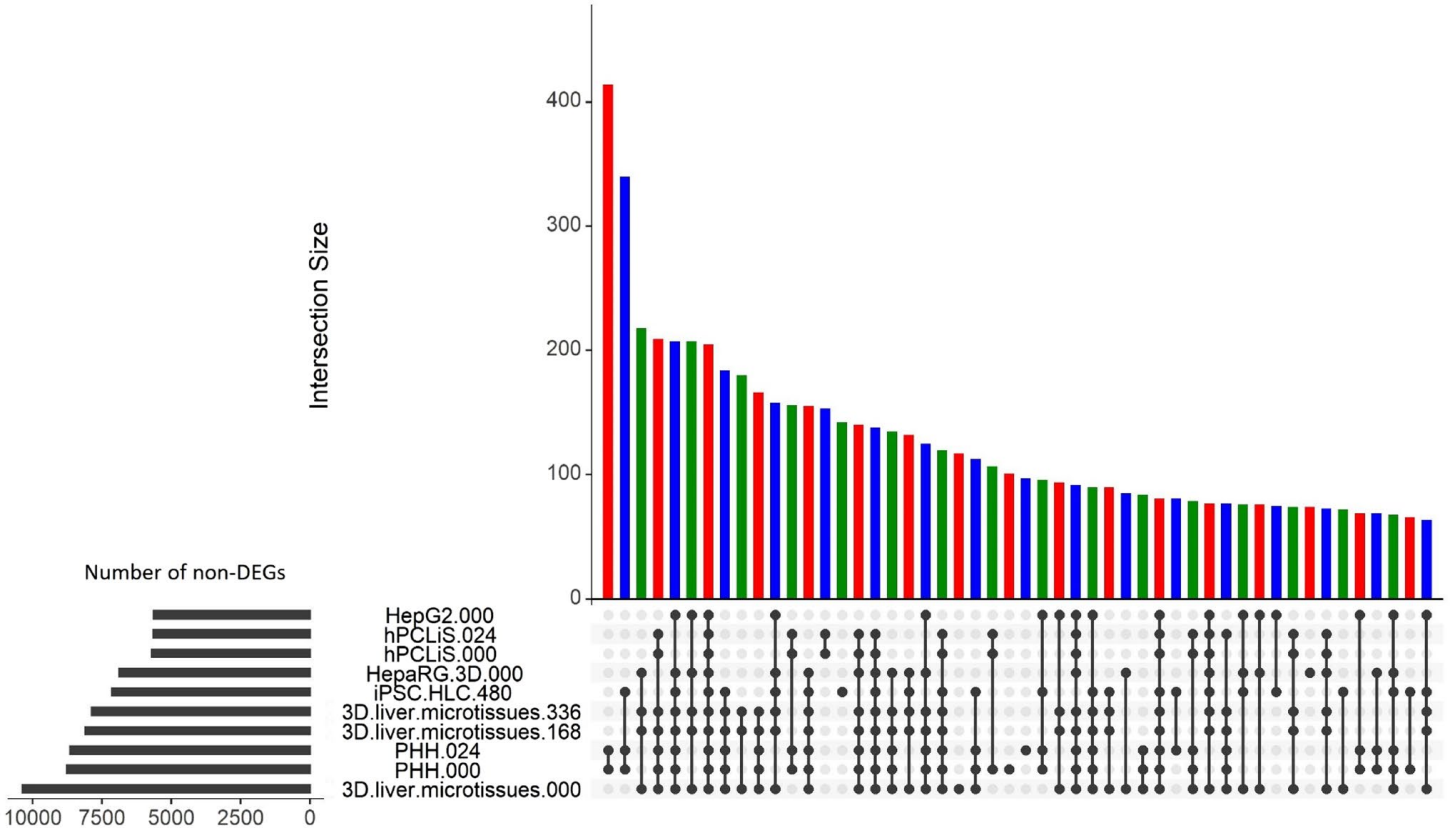
Overlap and number of non-DEGs. The number of non-DEGs for all cell models obtained after comparing against in vivo samples shown as horizontal bar plots on the left. The overlap between all cell models is shown as the main graph, top 50 overlaps are shown. For each cell model, the best three replicates were chosen as explained in the Bootstrapping section under Materials and methods. Different colors are used to enhance the readability of the graph

Normalized gene expression (mRNA profiles) of the in vitro cell models and in vivo liver were used to identify genes that are not differentially expressed (non-DEGs) (Fig. 4; Suppl. Table 3) to characterize which liver-like features the individual cell models possess. The numbers of non-DEGs of 3D liver microtissues (0 h) before cultivation was highest compared to the other cell models but dropped below the corresponding numbers of PHH after long-term cultivation for 168 and 336 h (Fig. 3). Similar numbers of non-DEGs were obtained for PHH before and after short-term cultivation for 24 h. The lowest numbers of non-DEGs were obtained for HepG2 and hPCLiS, while HepaRG and iPSC-HLC were intermediate. The highest overlap of non-DEGs was obtained between PHH before and after the cultivation period, illustrating that this system offers a relatively stable number of non-DEGs during short-term incubation for 24 h (Fig. 3). Moreover, a relatively large overlap of non-DEGs was obtained for iPSC-HLC and PHH.

**Fig. 5:**
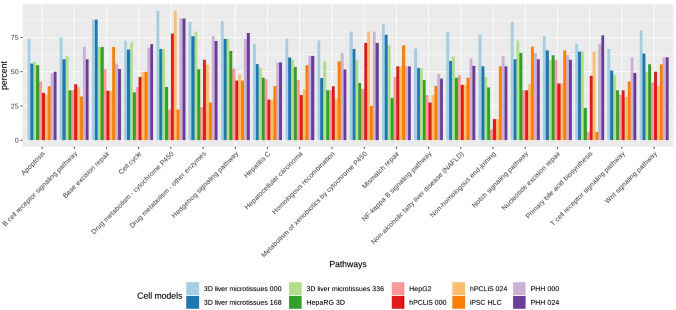
Pathway coverage of liver pathways by different cell models for the non-DEGs. The non-DEGs from all in vitro cell models were mapped onto important pathways in the liver for cell processes, regrowth and regeneration, cancer, viral infection, immune response, drug and xenobiotics metabolism, repair, and toxicity

To understand the effect on the biological processes, we then mapped all non-DEGs onto KEGG pathways (Suppl. Table 4a). Pathway mapping data can be used to study the specific processes/pathways of interest for each cell model and provide a metric of the similarity between liver tissue and the individual in vitro systems. Pathway coverage was calculated for the 20 liver pathways (Dufour et al. 2010) illustrated in Fig. 5 (Suppl. Table 5). Higher pathway coverage by non-DEGs implies higher similarity with the human liver.

3D liver microtissues (0 h) before the incubation period showed the highest coverage for most pathways but after 168 and 336 h of incubation, the coverage systematically dropped. In general, HepaRG 3D and HepG2 demonstrated a much lower coverage with HepG2 having the least. Exceptions were the high DNA repair functions of both tumor cell lines, with a relatively high coverage seen for base excision repair for HepaRG (68%) and nucleotide excision repair for HepG2 (59%). PHH showed a relatively high pathway coverage for all pathways and only small differences before (0 h) and after (24 h) after the cultivation period. For the primary bile acid biosynthesis pathway, PHH showed an increase during the 24 h cultivation period. For cytochrome P450 pathways, microtissues, PHH, and hPCLiS demonstrated a high coverage (metabolism of xenobiotics: hPCLiS 0 h: 71%, 24 h: 79%, PHH 0 h: 79%, 24 h: 71% and drug metabolism: hPCLiS 0 h: 78%, 24 h: 94%, PHH 0 h: 89%, 24 h: 89%), hence presenting their metabolizing capacities for exogenous substances. While the number of pathways for which hPCLiS exhibited a low coverage, was six for 0 h and eight for 24 h, it also had the highest coverage for two pathways for 24 h (both cytochrome p450 pathways). IPSC-HLC also showed a high coverage for DNA repair pathways with highest on base excision repair (68%) and nucleotide excision repair (66%). For other DNA repair pathways, iPSC-HLC also exhibited a higher coverage than HepaRG and HepG2 cell models.

### In vivo versus in vitro, using differentially expressed genes

**Fig. 6:**
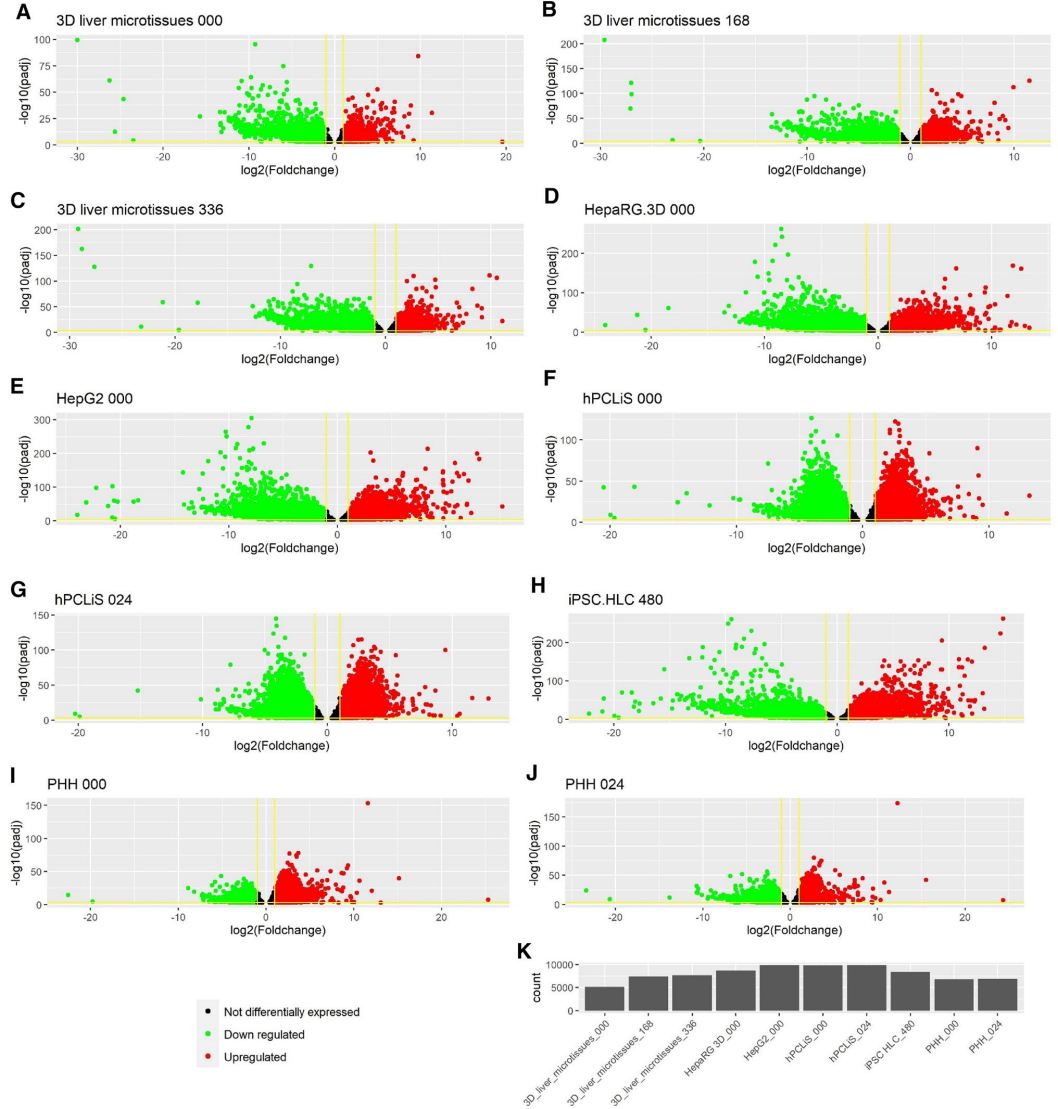
Volcano plots for DEGs. **a**–**j** The DEGs from various cell models when compared with healthy in vivo liver. The black dots represent not differentially expressed, green dots down regulated and red dots up-regulated genes. The x-axis is the log2foldchange of the gene expression between the healthy and in vitro cell models and y-axis is the p-adjusted (padj or *q* value). The horizontal yellow line corresponds to –log10 (0.05) where 0.05 is the threshold for padj and the vertical lines correspond to log2 foldchange <  − 1 and > 1. (K) Number of DEGs from each comparison

**Fig. 7:**
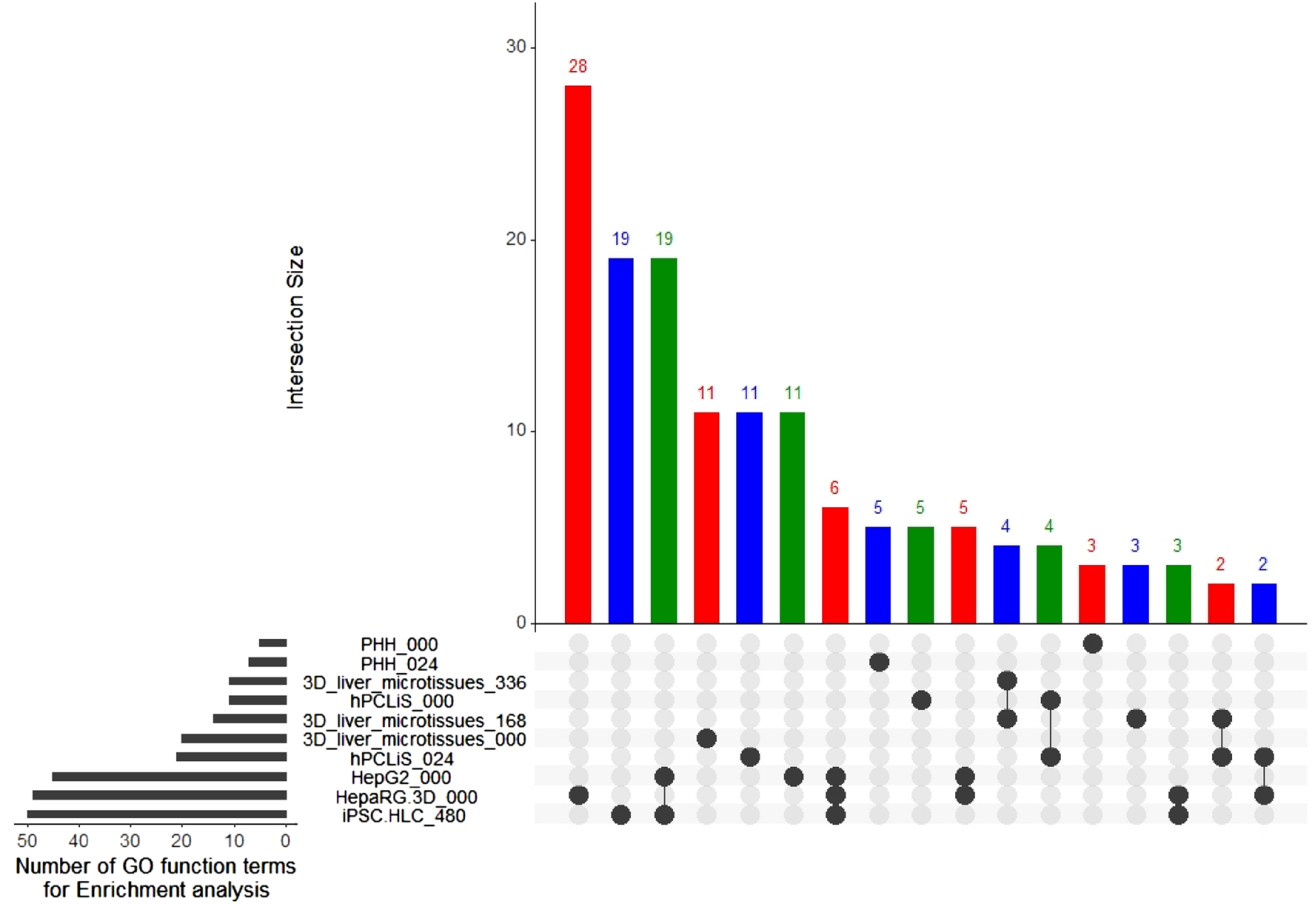
Overlap between the GO function for the DEGs. An enrichment analyses for the DEGs from all in vitro cell models was performed and the overlap for the resulting GO functions is presented. Different colors are used to enhance the readability of the graph

While the non-DEGs illustrated the similarities between the in vitro cell models and the in vivo liver, we also compared the differentially expressed genes (DEGs, *q* value (padj) < 0.05 and average counts > 10) to highlight the differences. The volcano plots demonstrated the extent of perturbation in the genes for all cell models (Fig. 6a–j). The number of DEGs were the highest for HepG2 (9910) and lowest for 3D liver microtissues 000 (5169) (Fig. 6k, Suppl. Figure 4). The number of DEGs were also high, comparable to HepG2, for hPCLiS both time points (0 h: 9837 and 24 h: 9890). The complete list of DEGs from all cell models is provided in Suppl. Table 6. The overlap between the DEGs from all cell models in Suppl. Figure 4 shows that the highest overlap was between all cell models except both time points from PHH. An enrichment analyses was performed for the DEGs using GOrilla (Eden et al. 2009) (Suppl. Table 7). While iPSC, HepaRG and HepG2 demonstrated the most perturbed GO functions, PHH had the least (Fig. 7). The highest overlap (19 GO functions) was between the iPSC and HepG2 cell models.

**Fig. 8:**
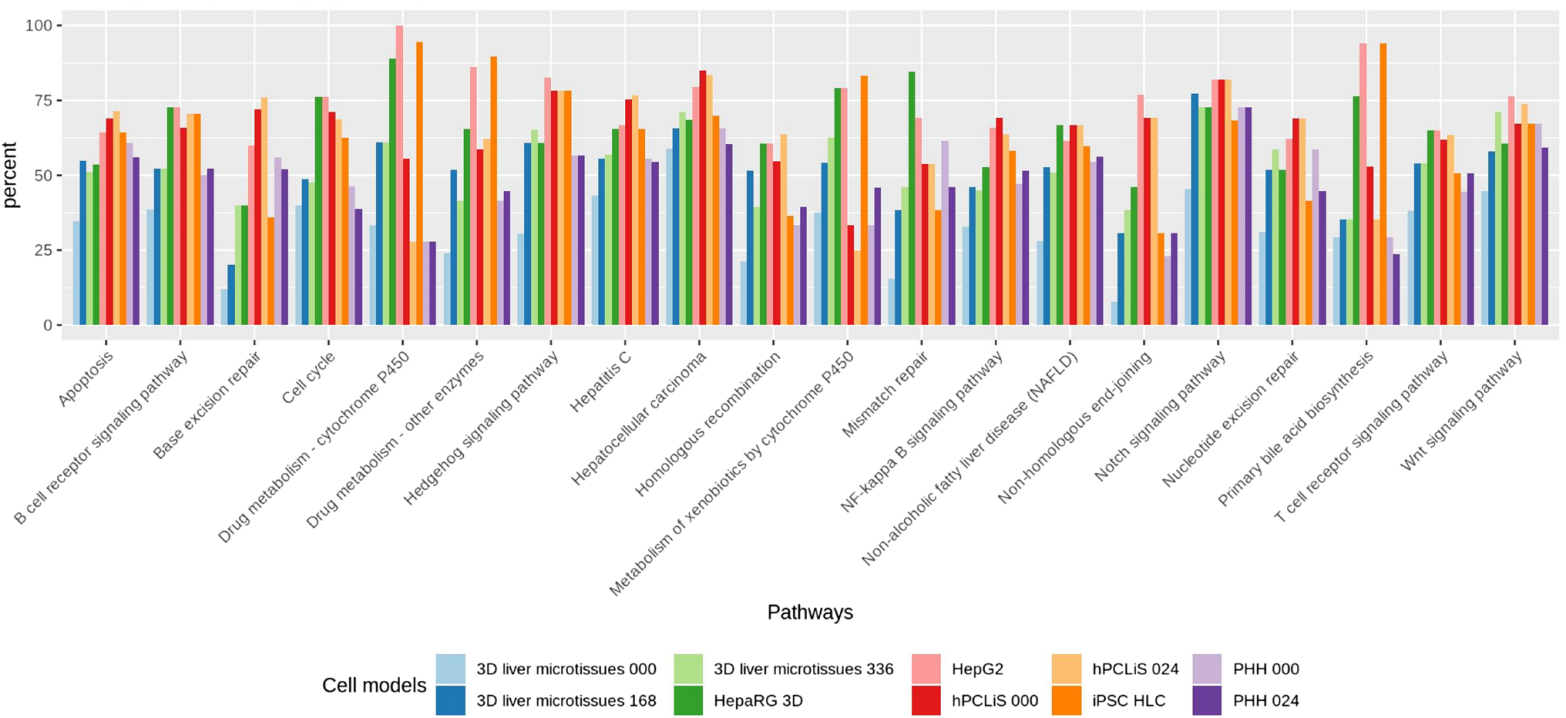
Pathway coverage of liver pathways by different cell models for the DEGs. The DEGs from all in vitro cell models were mapped onto important pathways in the liver for cell processes, regrowth and regeneration, cancer, viral infection, immune response, drug and xenobiotics metabolism, repair, and toxicity

The DEGs were also mapped onto the pathways to check their coverage (Fig. 8). A higher coverage by DEGs means that the cell models share low similarity with healthy in vivo liver. It is important to mention here that the pathway coverage for the DEGs is not the inverse of the pathway coverage of non-DEGs, this is because different genes can make similar proteins. The pathways are proteins interacting with each other and due to ambiguity in protein-gene relationships, pathway mapping tools, frequently map more than one gene to a protein. The pathway coverage of the DEGs illustrated an opposite mapping trend than non-DEGs (Fig. 5) and correctly so. Overall HepG2, HepaRG, and hPCLiS illustrated the highest coverage whereas PHH and 3D liver microtissues showed lowest coverage and iPSC-HLCs had high for some and low coverage for other pathways. Pathway mappings on DEGs from all cell models for all human pathways are provided in Suppl. Table 4b.

The changes in the expression of genes, differentially expressed genes, can be linked to the fluctuations in the expression of different transcription factors (TFs). The Network influence score (NIS), defined by the expression of downstream regulated genes, for the transcription factors from all the cell models was calculated using CellNet (Suppl. Figure 5a–g). These differences were calculated with respect to the cell/tissue profiles of the CellNet. The results show that the transcription factor ATF5 had the highest perturbation for all cell models except the PHH where it was shown to be the least perturbed. In the case of PHH, NR1H4 was the most affected factor. Moreover, in the case of PHH, ATF5 exhibited perturbation in the opposite direction than all other cell models. A similar analysis using the microarray data for freshly extracted hepatocytes, PHH and hiPSC using the microarray data illustrated a different list of TFs being affected. However, different types of data used for the two studies (microarray and transcriptomics) might be the reason for this difference.

**Fig. 9:**
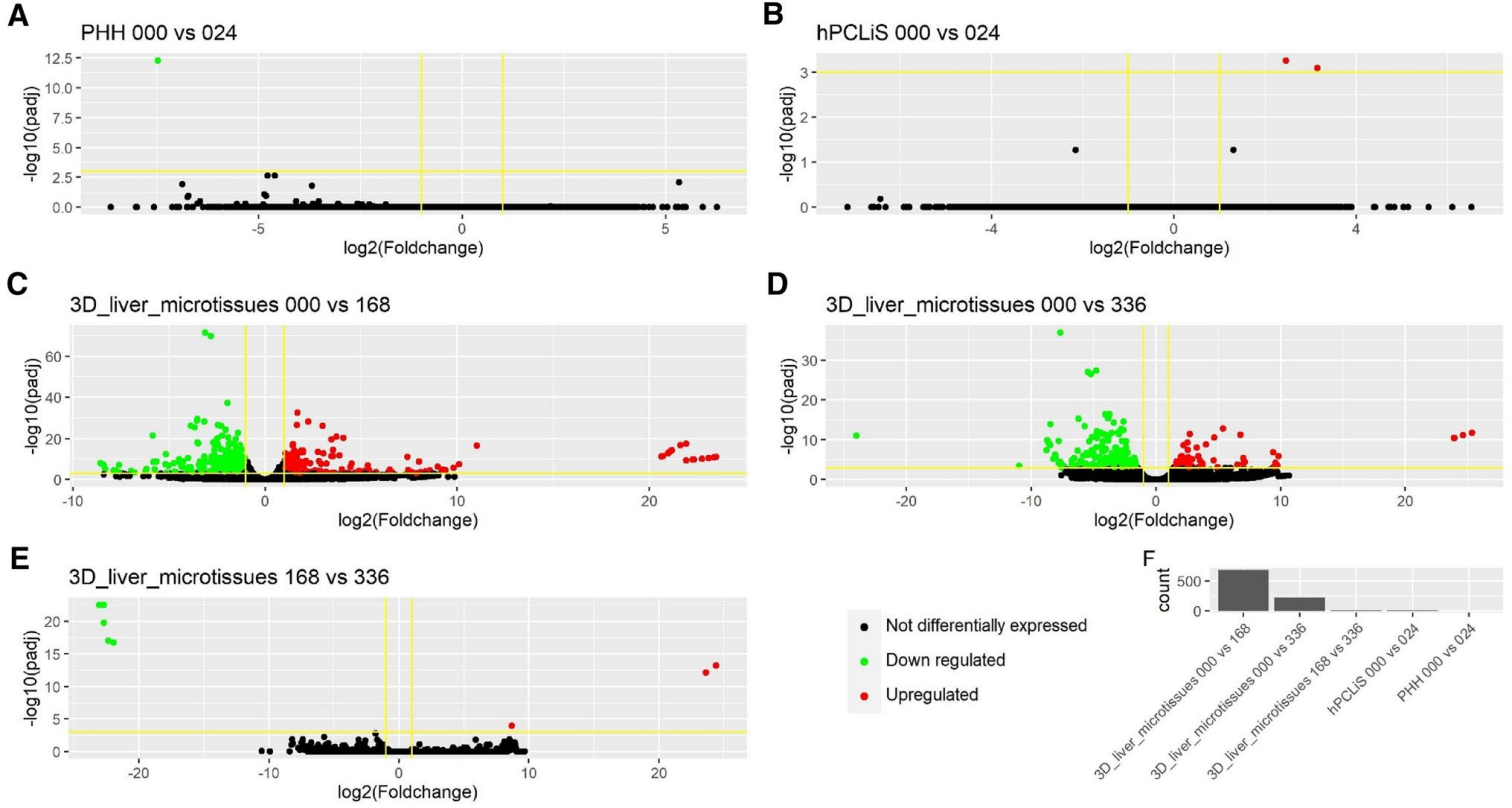
Volcano plots for differentially expressed genes (DEGs) from comparison of incubation periods for the cell models. RNA-Seq data was available for different time points for PHH (0 and 24 h), hPCLiS (0 and 24 h), and 3D liver microtissues (0, 168, and 336 h). DEGs were computed between different incubation times for each cell model. **a**–**e** Volcano plots for the DEGs computed for different comparisons. The black dots represent not differentially expressed, green dots down regulated and red dots up-regulated genes. The x-axis is the log2 foldchange of the gene expression between the healthy and in vitro cell models and y-axis is the p-adjusted (padj or *q* value). The horizontal yellow line corresponds to –log10 (0.05) where 0.05 is the threshold for padj and the vertical lines correspond to log2 foldchange <  − 1 and > 1. **f** Number of DEGs from each comparison

Furthermore, we also investigated how the cell models behaved over the incubation period. Three cell models, namely, PHH, hPCLiS, and 3D liver microtissues were incubated for different time durations. We computed the DEGs for each cell model over different time points (Fig. 9). PHH and hPCLiS show only very small variation in their expression profile over time, with a single gene differently expressed for PHH (0 h vs 24 h) and two DEGs for hPCLiS (0 h vs 24 h). 3D liver microtissues show a more important effect of time, with 684 DEGs between 0 and 168 h, 223 between 0 and 336 h and 8 for 168 h vs 336 h. While 3D liver microtissues illustrated comparatively higher number of DEGs, it should also be acknowledged that the incubation period for 3D liver microtissues was much longer than PHH and hPCLiS. PHH and hPCLiS that had same incubation period showed only a few genes perturbed over time.

### In vivo versus in vitro, using differential transcript usage

**Fig. 10:**
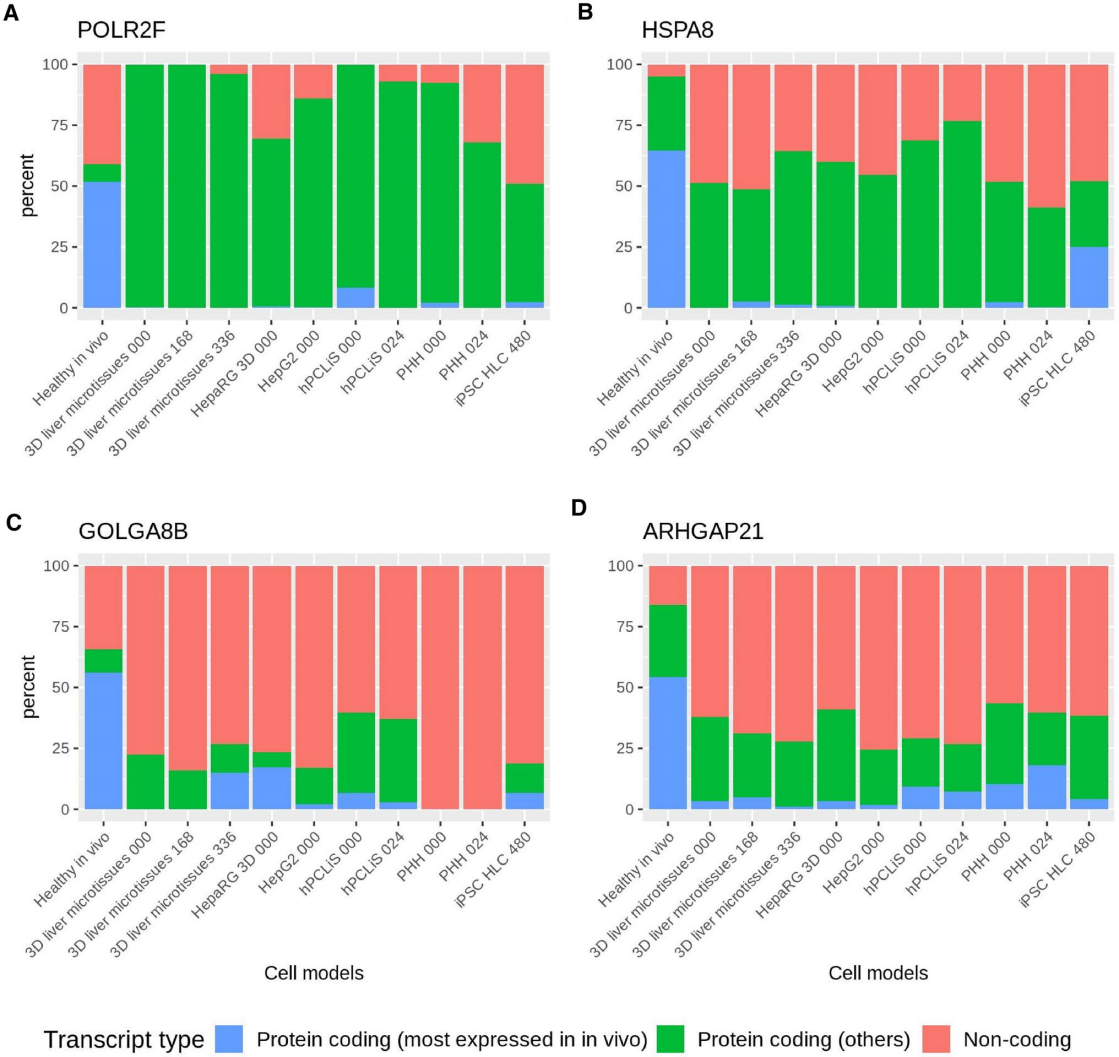
Examples of four non-DEGs that show major differential transcript usage (DTU). Transcript usage illustrated for four genes that were not differentially expressed at the gene level (non-DEGs) but had differential transcript usage (DTU). The most expressed protein coding transcript in vivo is replaced by other protein coding and/or non-coding transcripts **a** *POLR2F*, **b** *HSPA8*, **c** GOLGA8B, and **d** ARHGAP21

In the previous analyses, RNA-Seq data have been analyzed to identify differences between in vivo and in vitro cell models for the total gene expression generated by all isoforms of a gene. If the proportion of expression changes between different isoforms of a gene, total gene expression may remain constant. However, the different transcript usage (DTU) may nevertheless be relevant, because DTU may generate functionally different gene products. Differences in transcript expression (DTU) may be caused by alternative splicing, preference for one transcription start site over the other, spatial availability of transcription factors, and other elements. In standard RNA-Seq analysis, gene expression is assessed by summing the expression of all the transcripts for a given gene, and then it conceals the genes regulated at the splicing level. Genes with significant differential usage (*p* value < 0.01) at transcript level were then removed from the list of non-DEGs, giving the non-DEG^DTU−^ (Suppl. Table 8). An exhaustive list of DTU for all the cell models can also be found in the supplementary (Suppl. Table 9). The gene for which transcripts had differential usage (DTU) should be removed from the non-DEGs to fine-tune the analyses. This was illustrated for the examples of four genes that were non -differentially expressed but display DTU for the in vitro cell models (Fig. 10). The highest expressed protein coding transcript of *POLR2F* (DNA-directed RNA polymerases I, II, and III subunit *RPABC2*) was mostly replaced by other protein coding transcripts. For *GOLGA8B* (Golgin subfamily A member 8B) and *ARHGAP21* (Rho GTPase-activating protein 21), it was predominantly replaced by non-coding transcripts. *HSPA8* (Heat shock cognate 71 kDa protein) exhibited a different pattern, where the highest expressed protein coding transcript was replaced by other protein coding and non-coding transcripts. The highest expressed protein coding transcript in the case of *POLR2F* (52%) was reduced to < 2% in all cell models except hPCLiS 0 h (9%) while for *HSPA8*, it was reduced from 65 to < 3% for all except iPSC-HLC (25%). Similar trends can be seen for *GOLGA8B* and *ARHGAP21* (Fig. 10).

An investigation at this level revealed major changes in transcript usage for all in vitro cell models (Fig. 5). After removing the DTUs from the non-DEGs, termed as non-DEGs^DTU−^ (Fig. 3), their count and pathway coverage decreased. As for non-DEGs, a similar trend can be seen for non-DEGs^DTU−^ in terms of pathway coverage. Pathway mapping data of non-DEGs^DTU−^ for each cell model for all KEGG pathways are also provided to investigate queries per cell model and/or pathway (Suppl. Table 3b).

## Discussion

Different in vitro liver cell models have been developed for studying the effects of toxic compounds in humans. In the past, these models have been evaluated time and again for specific processes and components, giving a limited overview (Guillouzo et al. 2007; Soldatow et al. 2013; Wilkening et al. 2003), however, a systematic comparison of RNA-Seq data is not yet available. We compared these models at baseline gene expression using RNA-Seq. While in vivo and in vitro samples were sequenced on different platforms (Illumina NovaSeq 6000® and HiSeq 2000®, respectively), it is important to consider that both samples were produced using the same library preparation method, and both Illumina sequencers produced comparable results. The in vivo samples were sequenced with longer reads (150 bp) compared to cell samples (100 bp) but this cannot be expected to cause larger differences in the data as the read length higher than 50 bp does not drastically impact the outcome (Chhangawala et al. 2015; Rizzetto et al. 2017). Once a read’s position can be mapped unambiguously, longer reads do not add much value in a quantification-based analysis (Stark et al. 2019).

As expected, in comparison to the human in vivo liver, the highest pairwise spearman’s correlation was shown by the non-cancerous human liver-derived cell models, such as 3D liver microtissues and PHH. The cancer-derived cell models and iPSC-HLCs were still classified liver based on CellNet analysis but obtained the lowest classification scores using the human liver as reference. CellNet provides an easy and direct way to compare the cell models but it uses single-end (SE) reads for building the consensus expression profiles and GRNs (Radley et al. 2017) to accommodate more data available in public domains. However, SE sequences have poor coverage and low resolution of the 3′ end of the transcripts as compared to paired-end sequences. Thus, further approaches besides CellNet are required.

Therefore, we analyzed non-DEGs to focus on similarities between the in vivo and in vitro samples. Based on the number of non-DEGs and pathway coverage, 3D liver microtissues initially showed a high similarity with the liver in vivo but during the cultivation period of 168 and 336 h, the number of non-DEGs decreased. The largest deviation from the results obtained by CellNet was obtained with the hPCLiS and iPSC-HLC samples. Based on the results of CellNet, hPCLiS showed a high level of similarity with in vivo liver but using non-DEGs a relatively low resemblance was observed. On the other hand, for iPSC-HLC, CellNet predicted poor similarity but non-DEGs demonstrated a higher degree of similarity. The difference between results obtained by CellNet and analysis of non-DEGs could be due to differences in the level of lowly expressed genes which may gain more weight in the analysis of non-DEGs than in CellNet due to downsampling in CellNet (Radley et al. 2017). Furthermore, with DEGs, the highest similarity was observed for 3D liver microtissues and PHH, and lowest for HepG2 and hPCLiS 000 for the enrichment analysis and pathway coverage.

In addition to non-DEGs and DEGs, we also explored DTU thus highlighting the genes which are not differentially expressed on the gene level but exhibited significant differential usage of isoforms on the transcript level. The change in the amount of expression of the different transcript types in the cell models provided another metric to distinguish liver similar and dissimilar cell models. The analysis of the DTU first resulted in reduced numbers of non-differentially expressed genes (non-DEGs^DTU^) and hence pathway coverage. While globally the results of pathway coverage were similar to non-DEGs, studying the DTUs helped in identifying the genes which were differentially spliced between the in vivo liver and the in vitro system, notably by having a dominant protein coding or non-coding transcript(s). An important point worth mentioning here is that in our methodology, we used a stricter *p* value cut-off in the case of DTU (0.01), because isoform mapping is known to induce a higher false-positive rate (Rehrauer et al. 2013; Soneson et al. 2016). The evaluation of the DTUs aid in identifying the regulation control of expression of different protein coding and non-coding transcripts and conservation of the function of the proteins which otherwise remains oblivious at the gene level, as illustrated for the four exemplary genes in Fig. 7: these four genes are responsible for the process of transcription to protein trafficking and localization. First, *POLR2F* is a component of RNA polymerases I, II, and III which plays an important role in transcription (Kershnar et al. 1998), while *HSPA8* which is involved in a wide variety of cellular processes and also takes care of protein folding, transport, and proteolysis (Stricher et al. 2013). *GOLGA8B* and *ARHGAP21* are responsible for maintaining the Golgi apparatus (Dubois et al. 2005; Sousa et al. 2005) and were shown to be differentially expressed at the transcript level. The differential expression of these genes implies that the functions of the Golgi (modifying, sorting, and packaging of proteins for secretion) may be perturbed. The present results show that the 3D liver microtissues (0 h) demonstrate a particularly high Spearman correlation, CellNet classification score, GRN status, number of non-DEGs, non-DEGs^DTU−^, and pathway coverage. During the cultivation period, these values decrease. It should, however, be considered that cultivation periods of 168 and 336 h are relatively long and it is difficult to maintain in vivo like properties for such long periods. For short-term incubation of 24 h PHH represent an adequate system, since the CellNet classification score, GRN status, number of non-DEGs, non-DEGs^DTU−^, and pathway coverage remained almost unchanged during the cultivation period. Therefore, in agreement with previous studies (Grinberg et al. 2014, 2018), cultivated primary hepatocytes seem to represent an adequate system for short-term experiments to identify genome-wide expression changes. Over the incubation period, the non-DEG pathway coverage for primary bile acid biosynthesis increased for PHH. This is in agreement with previous studies showing that isolated hepatocytes establish bile canaliculi and express bile acid excretion carriers at their apical membranes during the first 24 h in culture (Godoy et al. 2016; Reif et al. 2015). While the hPCLiS cell models exhibited lower similarity with in vivo liver compared to microtissues and PHH, this may be explained by the location of extraction of the tissue from the liver cancer patients.

HepG2 cells lost numerous functions compared to primary hepatocytes. Nevertheless, they are used for in vitro studies as they represent a relatively inexpensive, easy to handle cell line. These present a higher intermodal variability, probably because the cancer cells under uncontrolled cell division accumulate mutations over time. The same holds for HepaRG cells that still show slightly more non-DEGs than HepG2 cells but are less similar to in vivo liver tissue as PHH or microtissues. Several recent studies reported that HepaRG 3D models mimic in vivo liver (Ott et al. 2017; Ramaiahgari et al. 2017; Takahashi et al. 2015) but these studies did not perform an RNA-Seq based comparison to human liver tissues.

The use of human iPSCs as a renewable source for the generation of human hepatocytes holds great promise as non-transformed hepatocytes from individuals with multiple genetic backgrounds could be generated. However, consistent with other publications (Godoy et al. 2015, 2018; Heslop and Duncan 2019), we here found that iPSC-derived hepatocyte-like cells still show major differences compared to liver tissue and primary human hepatocytes. CellNet analysis of the RNA-seq expression profiles of human-iPSC-HLCs demonstrated that the iPSC progeny shows a low CellNet classification score for the human liver. Moreover, they still share a resemblance with embryonic stem cells and exhibit some overlap with the expression profiles of the intestine and colon cells/tissue (Suppl. Table 2), as previously described in other studies (Godoy et al. 2015). Additionally, non-DEGs and non-DEG^DTU−^ were identified by comparing the mRNA profiles of the iPSC-HLCs with in vivo liver expression data. With around 7118 non-DEGs and 7087 non-DEG^DTU−^ iPSC-HLCs demonstrated an even higher similarity to human liver tissue than hPCLiS, HepaRG, and HepG2 but ranged clearly behind microtissues and PHH. However, when mapping onto liver pathways selected from KEGG, the iPSC-HLCs showed only a relatively low pathway coverage. Taken together, the results illustrate that iPSC-derived cells performed better than the cancer models (HepG2 and HepaRG) and in some cases even better than hPCLiS as well. Though these results suggest that they exhibit some similarity to in vivo liver, there are still significant hurdles to overcome before iPSC-derived hepatic progeny reach a high similarity to real hepatocytes. Different strategies to improve HLC differentiation may include chemical engineering of the culture media (Siller et al. 2015), the use of 3D organoid cultures and microfluidic systems to recreate the in vivo hepatocyte niche and to allow the manipulation of oxygen gradients and the delivery/removal of specific factors (Ong et al. 2017; Takebe et al. 2013). In addition, as several TFs are highly differentially expressed between iPSC-HLCs and in vivo liver, another way to improve iPSC-HLCs maturation could be by up/downregulation of these misregulated TFs (Suppl. Figure 5f) (Godoy et al. 2015, 2018). It is important to consider that these results were obtained from baseline comparisons and, while analyzing or deriving hypothesis from these results, it should be kept in mind that their response to stress and/or exposure to chemicals still has to be elucidated.

## Electronic supplementary material

Below is the link to the electronic supplementary material.

Supplementary file1 (DOCX 3309 kb)

Supplementary file2 (DOCX 1229 kb)

Supplementary file3 (DOCX 89 kb)

Supplementary file4 (DOCX 254 kb)

Supplementary file5 (DOCX 39 kb)

Supplementary file6 (DOCX 324 kb)

Supplementary file7 (DOCX 312 kb)

Supplementary file8 (DOCX 316 kb)

Supplementary file9 (DOCX 330 kb)

Supplementary file10 (DOCX 341 kb)

Supplementary file11 (DOCX 326 kb)

Supplementary file12 (DOCX 90 kb)

Supplementary file13 (DOCX 15 kb)

Supplementary file14 (DOCX 16 kb)

Supplementary file15 (DOCX 6068 kb)

Supplementary file16 (DOCX 127015 kb)

Supplementary file17 (DOCX 6727 kb)

Supplementary file18 (DOCX 31 kb)

Supplementary file19 (DOCX 668 kb)

Supplementary file20 (DOCX 272 kb)

## Data Availability

The raw data can be assessed from ENA: In vivo liver: PRJEB35350/ERP118386, PHH: PRJEB23590/ERP105351, iPSC-HLC: PRJEB23620/ERP105382, 3D liver microtissues: PRJEB24482/ERP106310, HepG2: PRJEB24466/ERP106294 and PRJEB24464/ERP106292, HepaRG 3D: PRJEB24487/ERP106315, and hPCLiS: PRJEB24484/ERP106312.
