# Supplementary material for: Comparing in vitro human liver models to in vivo human liver using RNA-Seq
Source: Arch Toxicol. 2020 Oct 27;95(2):573–89. doi: 10.1007/s00204-020-02937-6 (PMC7870774; doi:10.1007/s00204-020-02937-6)
Supplement: Supplementary file 1 — Supplementary file1 (DOCX 3309 kb) [file 204_2020_2937_MOESM1_ESM.docx]

1. **Supplementary materials and methods**
2. **Healthy liver *in vivo***

Control human liver samples were obtained from the Biobank Hospital La Fe that belongs to the Spanish National Biobank Network. Most of these livers come from donors who were primarily assigned to liver transplantation but due to failings in some of the inclusion criteria were donated to research. Studies involving human samples were performed in agreement with the Declaration of Helsinki and with local and national laws. The Human Ethics Committee of the Hospital La Fe Valencia approved the study procedures, and families’ informed consent was obtained by the National Transplant Organization Coordination. The anthropometric and analytical characteristics of these liver donors have been described elsewhere [[1](#_ENREF_1)].

1. **PHH**

Primary human hepatocytes were cultivated according to a published standard operation procedure [[2](#_ENREF_2)].

1. **iPSC derived hepatocyte-like cells**

The human induced pluripotent stem cell lines (hiPSCs or iPSC) SBAD2 and SBAD3 were purchased from StemBancc. Both iPSC lines were maintained on human matrigel (BD Biosciences) coated plates in E8 Flex medium (Gibco) in a humidified 5% CO2 incubator at 37°C. The iPSCs were differentiated towards hepatocytes following a previously published 20-day differentiation protocol with minor adjustments [[3](#_ENREF_3), [4](#_ENREF_4)]: namely, dimethyl sulfoxide (DMSO) was added from day 0 until day 12 at a concentration of 0.6% and increased to 2% from day 12 of differentiation onwards until the end of the hepatocyte differentiation. Additionally, during the first four days of differentiation, the cells were kept in hypoxic conditions.

1. **3D liver microtissues**

All spheroid 3D liver microtissues used in this study were 3D InSight™ Human Liver Microtissues (InSphero AG, Schlieren, Switzerland) and produced according to a patent-pending protocol (WO2015/158777A1) using Akura™/96 ultra-low attachment plates. 3D InSight™ Human Liver Microtissues were produced of human primary hepatocytes (lot YFA, BIOIVT, Westbury, NY) in co-culture with NPCs (lot EFC, BIOIVT). Single 3D liver microtissues in each well were incubated at 37 °C in a humidified 5% CO2 cell-culture incubator in BSA-free 3D InSight™ hLiMM TOX medium for 7, 14 and 21 days with regular medium exchange every 3-4 days. At each time point, 60 3D liver microtissues were harvested, washed in PBS, and dissolved in Trizol™ (Thermofisher) for total RNA extraction according to the manufacturer’s protocol.

1. **Culture of HepG2 cells**

HepG2 cells were cultured in Dulbecco's Modified Eagle's Media (DMEM) supplemented with 10% fetal bovine serum, 50U penicillin/mL, and 50μg streptomycin/mL. For subculturing purposes, cells were detached by treatment with 0.25% trypsin/0.02% EDTA at 37ºC.

1. **HepaRG 3D**

**Materials:**

Cell culture medium, medium supplements, and compounds were obtained from Sigma-Aldrich (UK) or Life Technologies (UK) unless stated otherwise.

**Cell culture:**

HepaRG cells (Cryopreserved differentiated) were obtained from Caltag Medsystems (UK) and were used for the formation of 3D spheroid cultures. Cells were seeded into ultra-low attachment (ULA) 96-well plates (Corning, USA) at a density of 2,000 viable cells per well and left overnight. HepaRG spheroids were seeded in 100 μl DMEM containing hepatocytes bullet kit (Lonza, UK) supplemented with 2 mM ultra glutamine, 25mM HEPES, and 10% FBS. HepaRG cells spontaneous self-aggregate into spheroids. Following 4 days after seeding, visible compact spheroids had formed, 50% of the medium was exchanged daily for serum-free medium (Williams E medium supplemented with 2 mM L-glutamine, 100 units/ml penicillin, 100 μg/ml streptomycin, 10 μg/ml insulin, 5.5 μg/ml transferrin, 6.7 ng/ml sodium selenite, 100 nM dexamethasone, and 10% FBS; Bell et al., 2016). Cells were maintained in a humidified atmosphere incubator with 5% CO2 at 37°C [[5](#_ENREF_5)].

**Transcriptomic expression profiling cell lysate generation**

In brief, at day 7 cells were washed in calcium and magnesium-free 1X PBS. With all residual PBS removed, 2X TempO-Seq lysis buffer (BioSpyder Technologies, proprietary kit) was diluted to 1X with PBS and added at a volume of 1µl per 1000 cells with a minimum of 10µl per well and incubated for 10 minutes at room temperature. Following lysis, the samples were frozen at -80°C.

1. **hPCLiS**
2. Human liver tissue

Human liver tissue was obtained from patients undergoing partial hepatectomy due to primary or secondary liver cancer. All patients prospectively consented to donate liver tissue for research. Background information on tissue donors is provided in table 1. Directly after resection, liver tissue was stored in ice-cold oxygenated Krebs-Henseleit buffer (KHB) at pH 7.42 (supplemented with 25 mM NaHCO_3_ and 2.5 mM CaCl_2_·H_2_O, all purchased from Sigma–Aldrich GmbH, Taufkirchen, Germany) and kept on ice until further preparation. For oxygenation, the buffer was saturated with Carbogen® LAB (95% O_2_, 5% CO_2_, Linde Gas GmbH, Stadl-Paura, Germany).

Suppl. Table 8: Donor characteristics

| **#** | **Age** | **Sex** | **Diagnosis** |
| --- | --- | --- | --- |
| 1 | 77 | Male | Hepatocellular adenoma |
| 2 | 64 | Female | Colorectal liver metastases |
| 3 | 70 | Male | Hepatocellular carcinoma |
| 4 | 63 | Female | Liver metastases from breast cancer |

1. Preparation and culture of human precision-cut liver slices (hPCLiS)

PCLiS were prepared and cultured as described previously by Granitzny et al. (2017) [[6](#_ENREF_6)] with minor modifications. In brief, cylinders with a diameter of 8 mm were punched from the whole tissue and collected in ice-cold oxygenated KHB. These Liver cores were sliced with a Krumdieck tissue slicer MD6000 (Alabama Research and Development, Munford, Alabama, USA) in ice-cold oxygenated KHB. Liver slices (Ø 8 mm, ~280 µm thickness) were then stored in ice-cold oxygenated KHB until incubation (see Fig. 1A – D).

William’s Medium E (WME) + GlutaMAX™ (Life Technologies, Darmstadt, Germany) supplemented with 25 mM glucose (Life Technologies, Darmstadt, Germany) and 50 µg/mL gentamicin (Life Technologies) was used as culture medium. Immediately after slicing procedure, slices were transferred into 6-well plates (3 slices/well) filled with 6 mL pre-warmed and oxygenated WME (see Fig. 1E). PCLiS were then pre-incubated for 1 h in carbogen-gassed culture boxes within an incubation shaking (80 rpm) cabinet Certomat® CT Plus (Satorius Stedim Systems GmbH, Göttingen, Germany) with an atmosphere of 37 °C and 90 % humidity (see Fig. 1F). After preincubation hPCLiS were transferred to new plates with fresh oxygenated medium and subsequently incubated under the same conditions for 24 h. For detailed information on the experimental protocol and gassing regime refer to Figure 2.

1. Sampling and storage in TRIzol™

Freshly prepared and cultured slices were transferred into RNase-free reaction tubes (1 slice/tube) filled with 1 mL TRIzol™ reagent (Thermo Fisher Scientific, MA, USA) and immediately homogenized for 20 s with an Ultra-Turrax® T8 (IKA®-Werke, Staufen, Germany). Samples were then stored at -80°C until shipping.


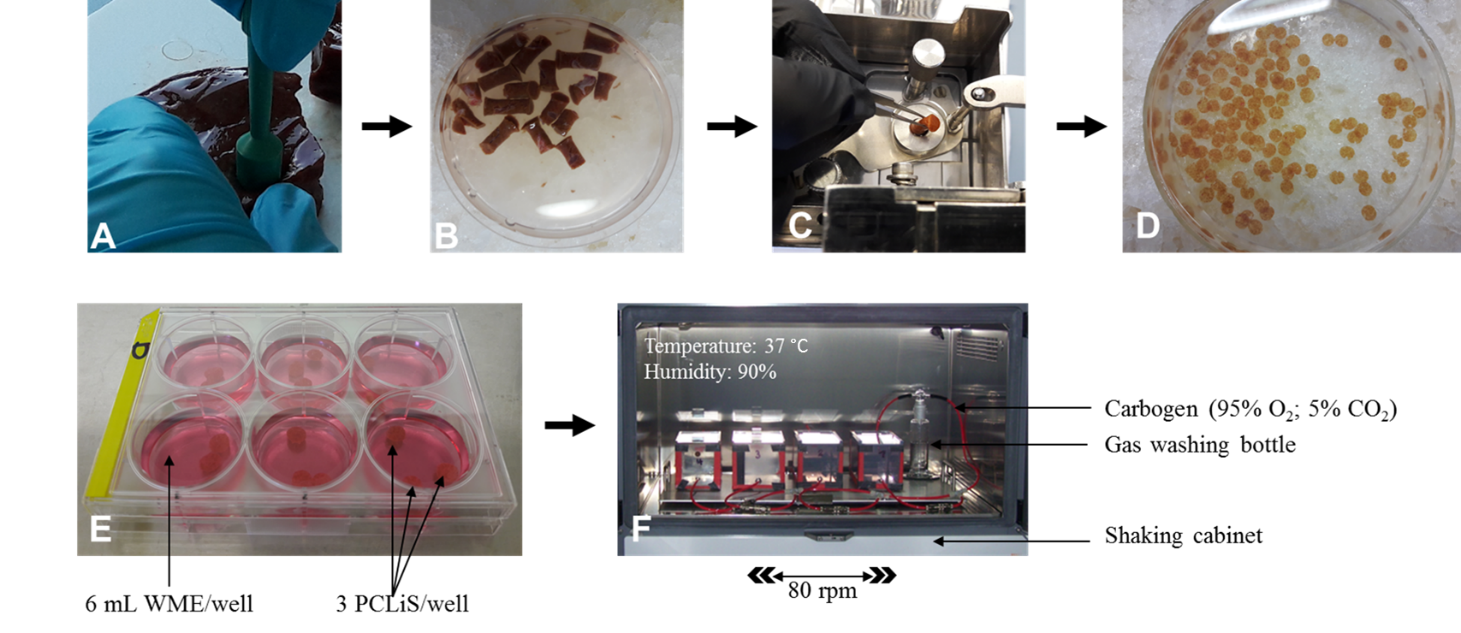


Suppl. Figure 6: Preparation and culture of hPCLiS


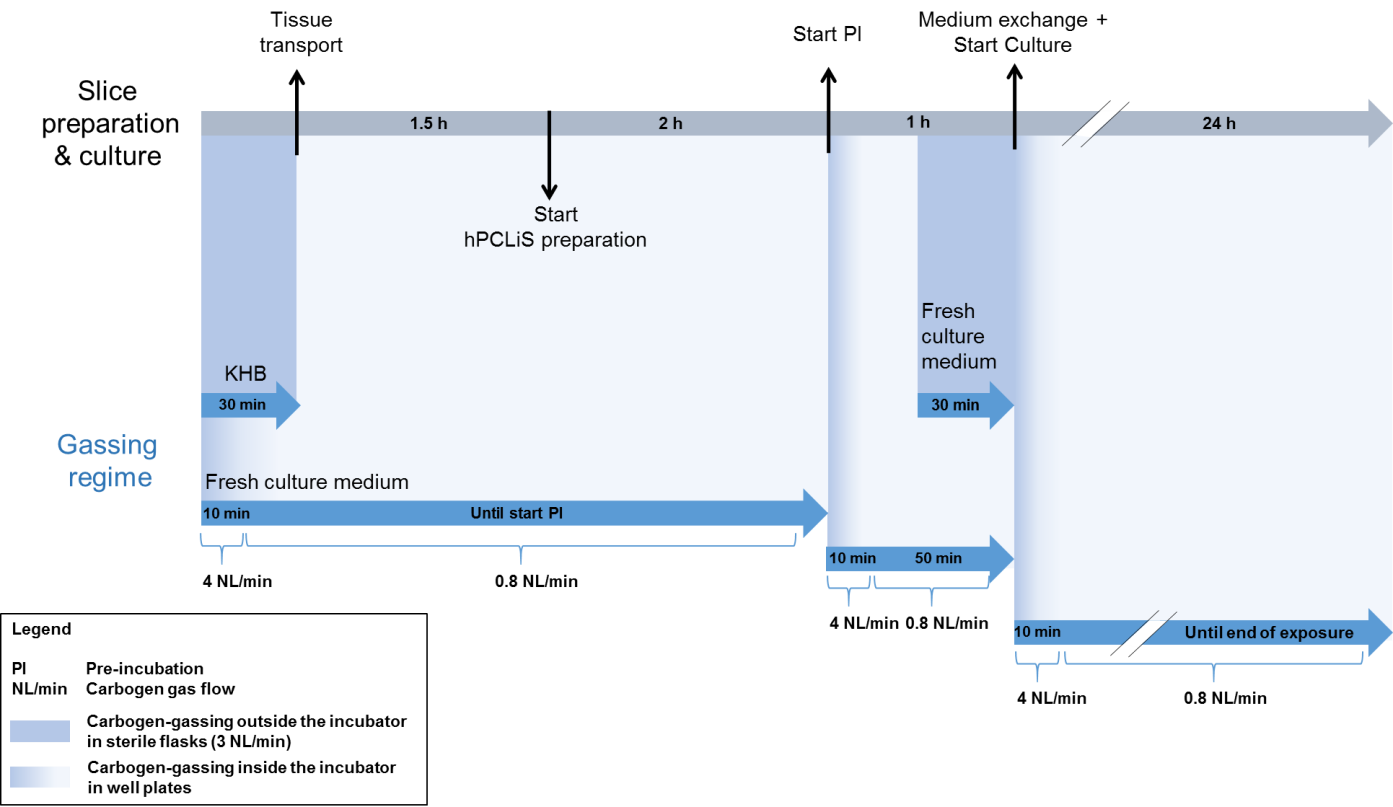


Suppl. Figure 7: Time schedule of hPCLiS preparation, culture and gassing regime.

1. **Supplementary Figures**


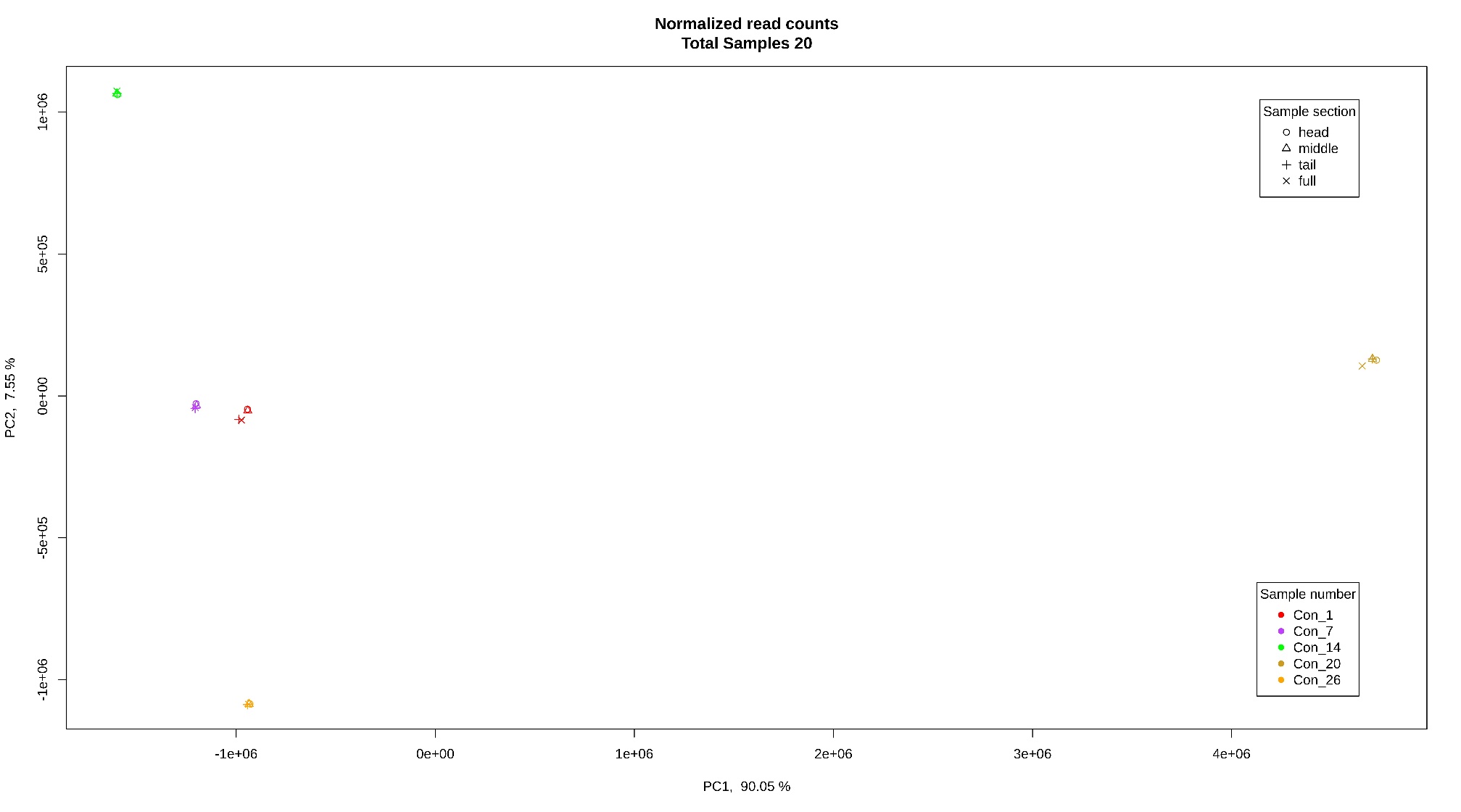


Suppl. Figure 1: PCA for the full, head, mid, and tail of the fastq files, demonstrating the similarity between the samples.

(a)
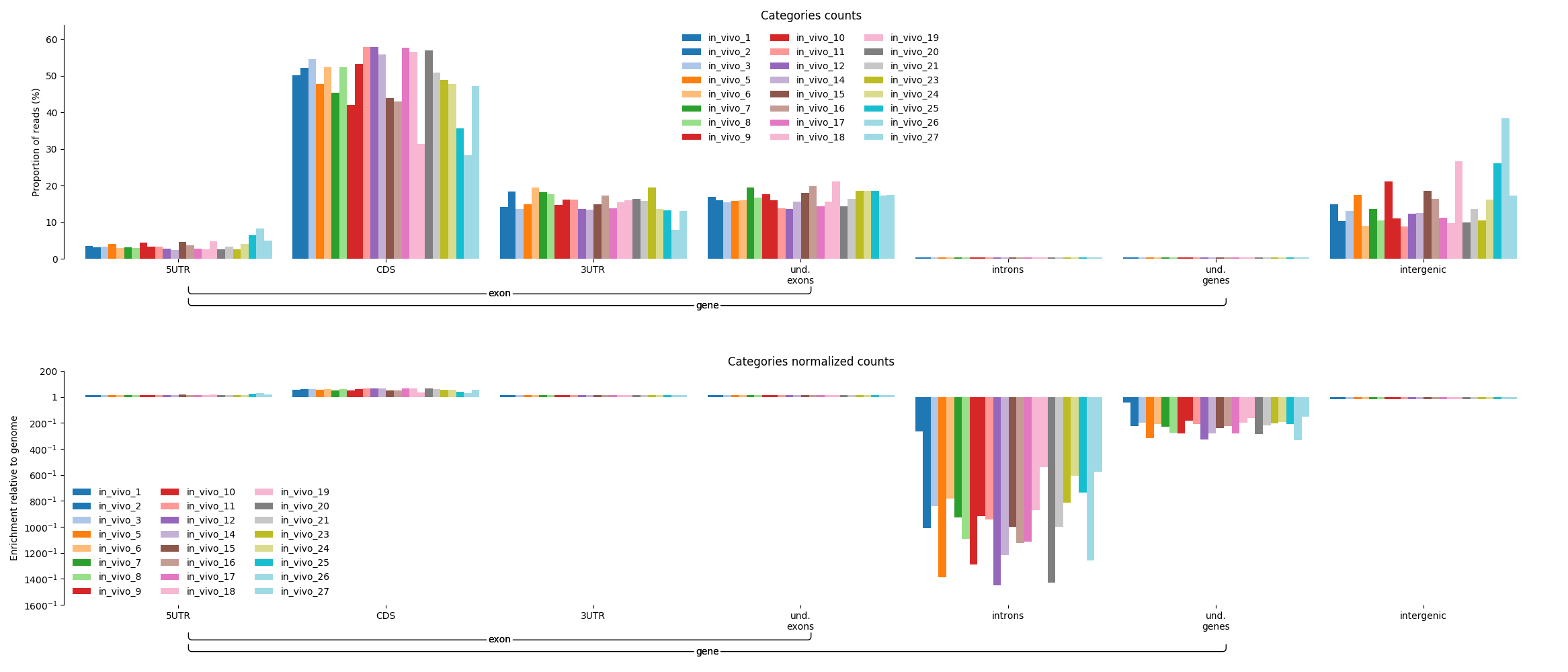


(b)
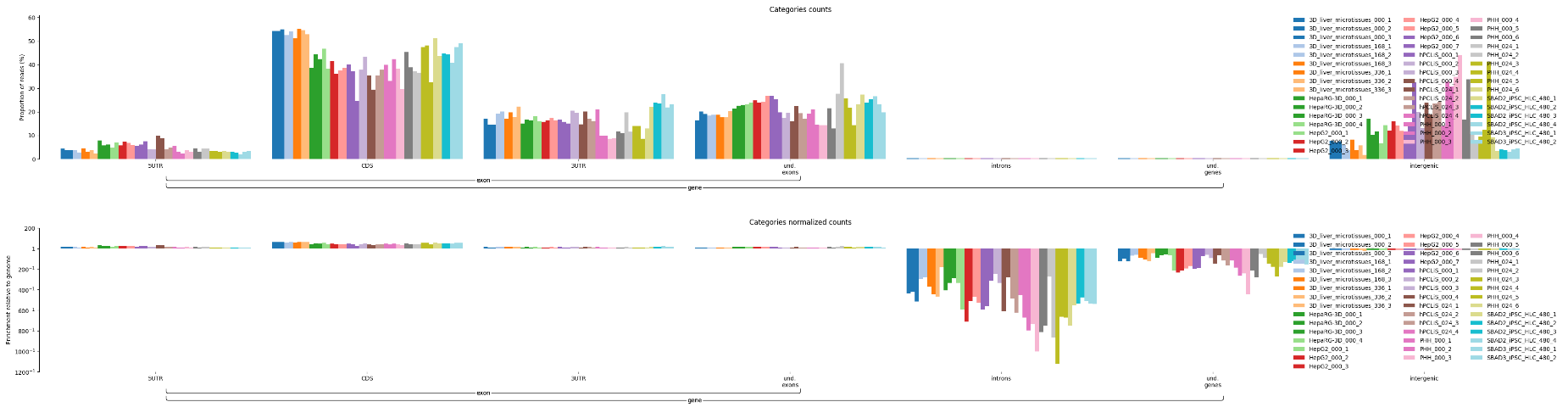


Suppl. Figure 2: Annotation of reads assessed by ALFA (a) *in vivo* healthy samples, (b) *in vitro* cell models.


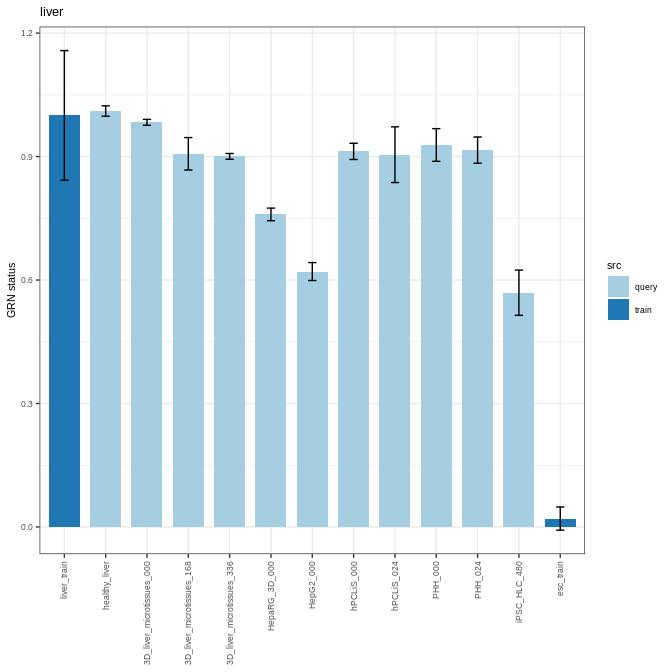


Suppl. Figure 3: GRN (Gene Regulatory Network) score calculated using R package CellNet [[7](#_ENREF_7)] for all the cell models.


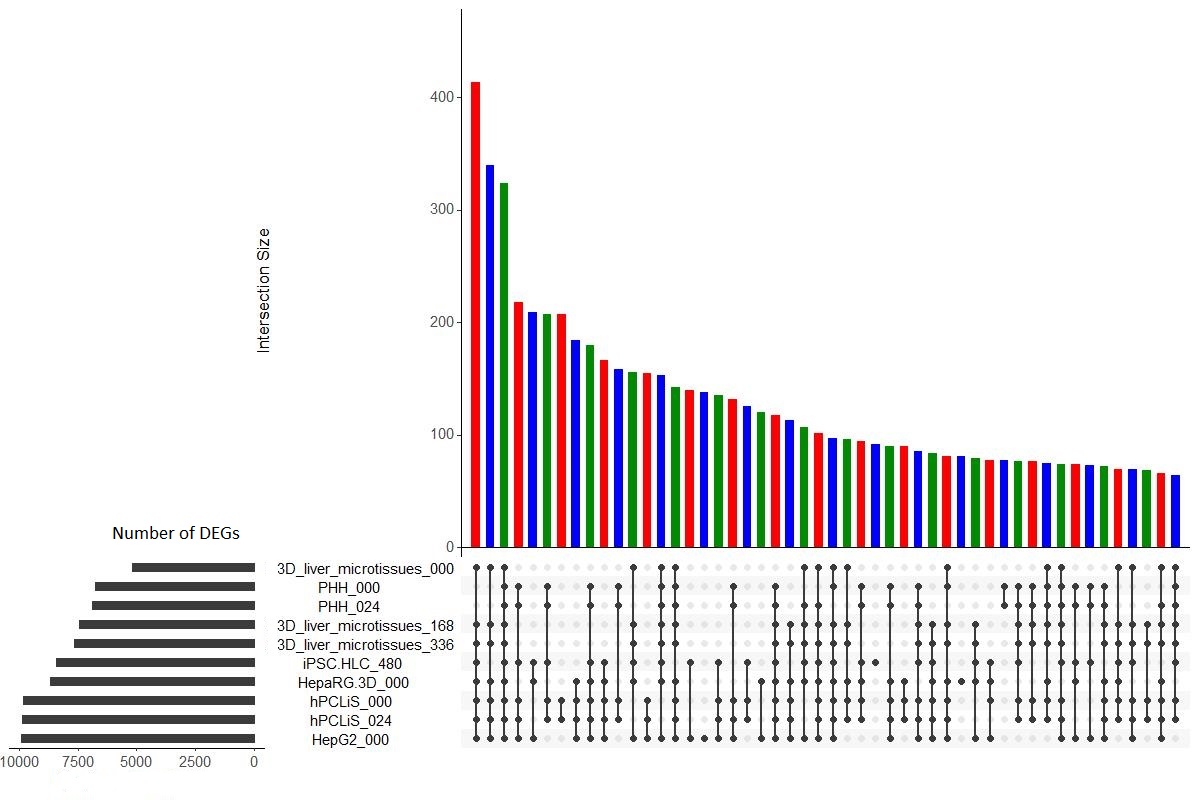


Suppl. Figure 4: **Overlap and number of DEGs**. The number of DEGs for all cell models obtained after comparing against *in vivo* samples shown as horizontal bar plots on the left. The overlap between all cell models is shown as the main graph, top 50 overlaps are shown. For each cell model, the best three replicates were chosen as explained in the Bootstrapping section under Materials and methods in the main paper.

(a)
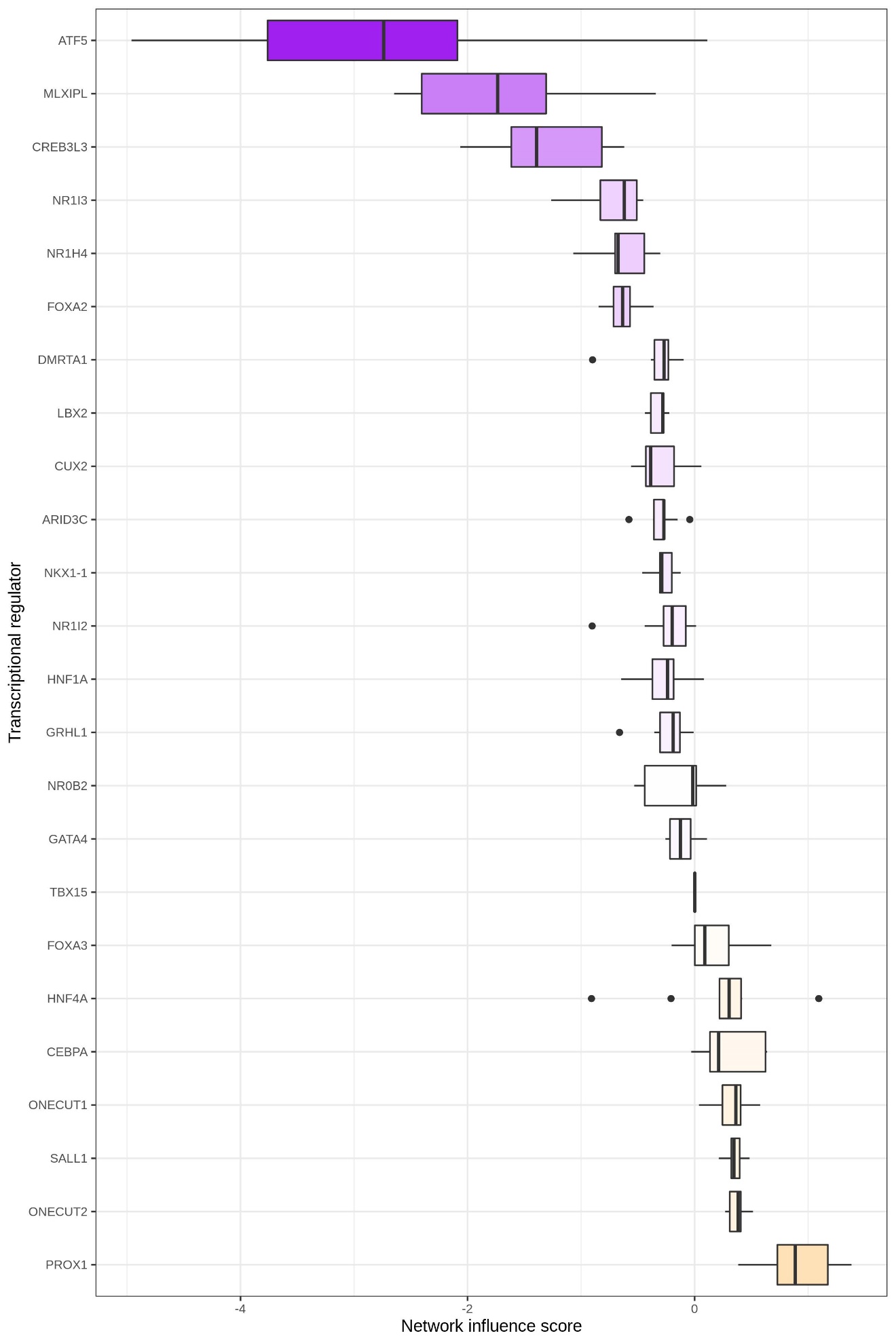


(b)
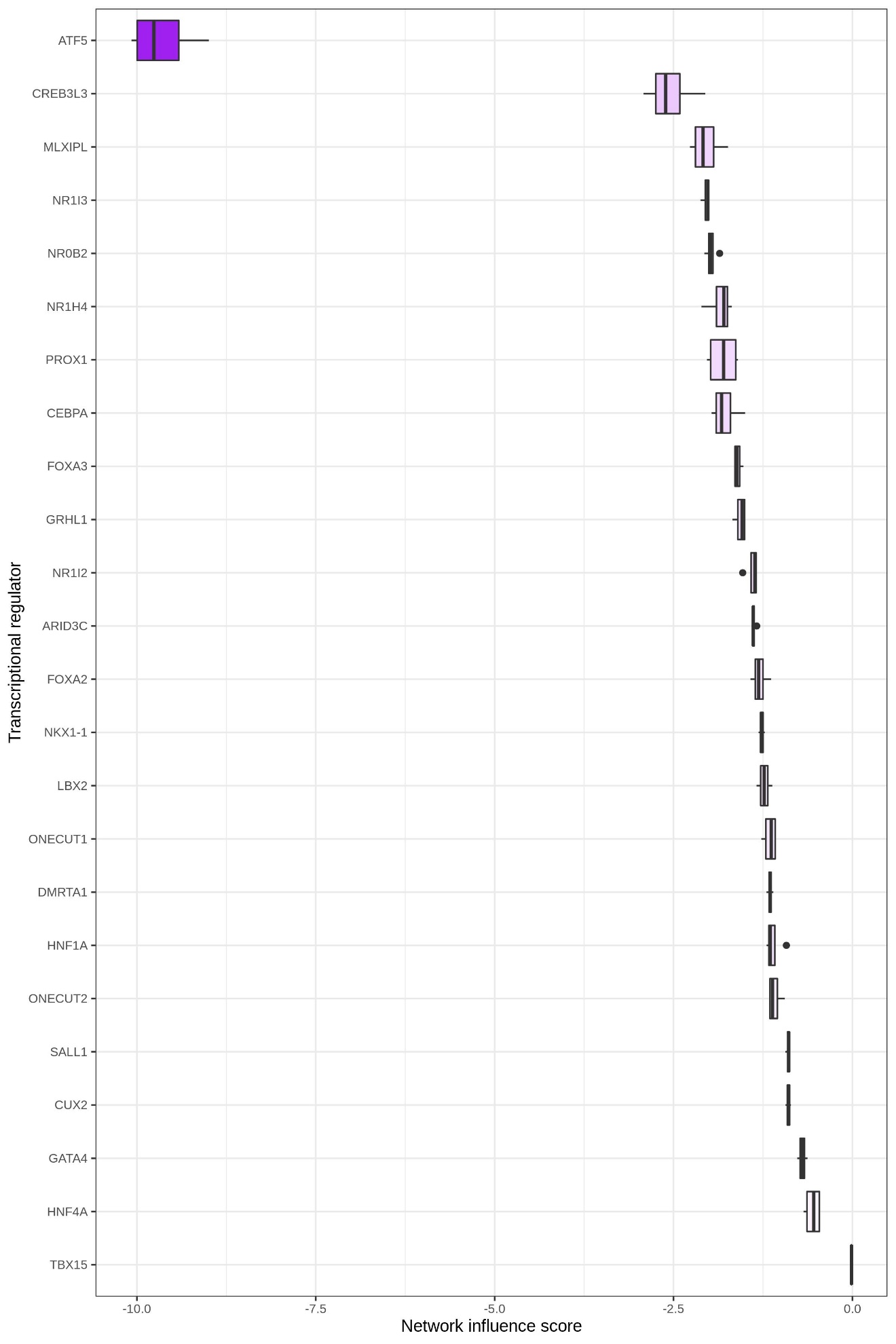


(c)
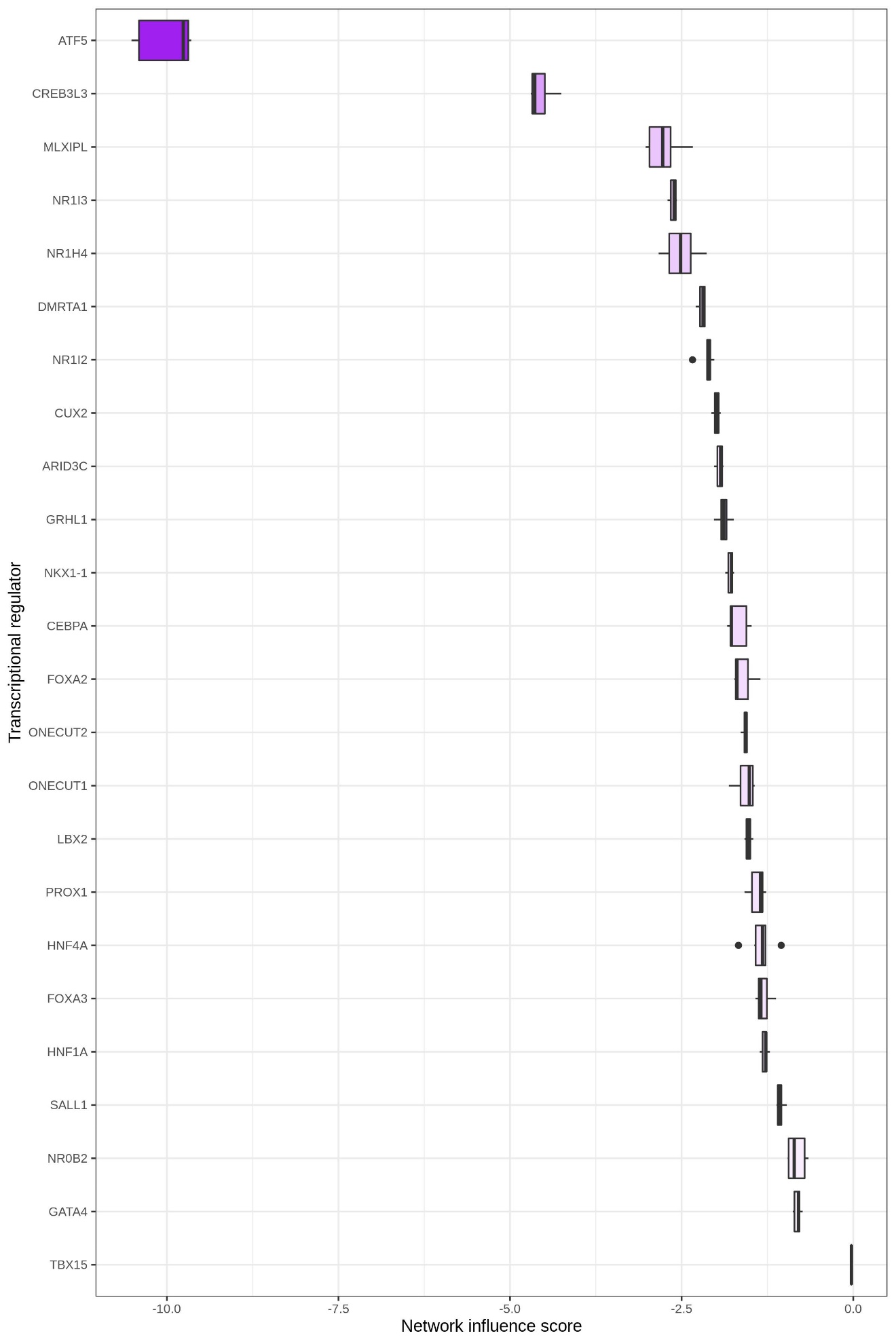


(d)
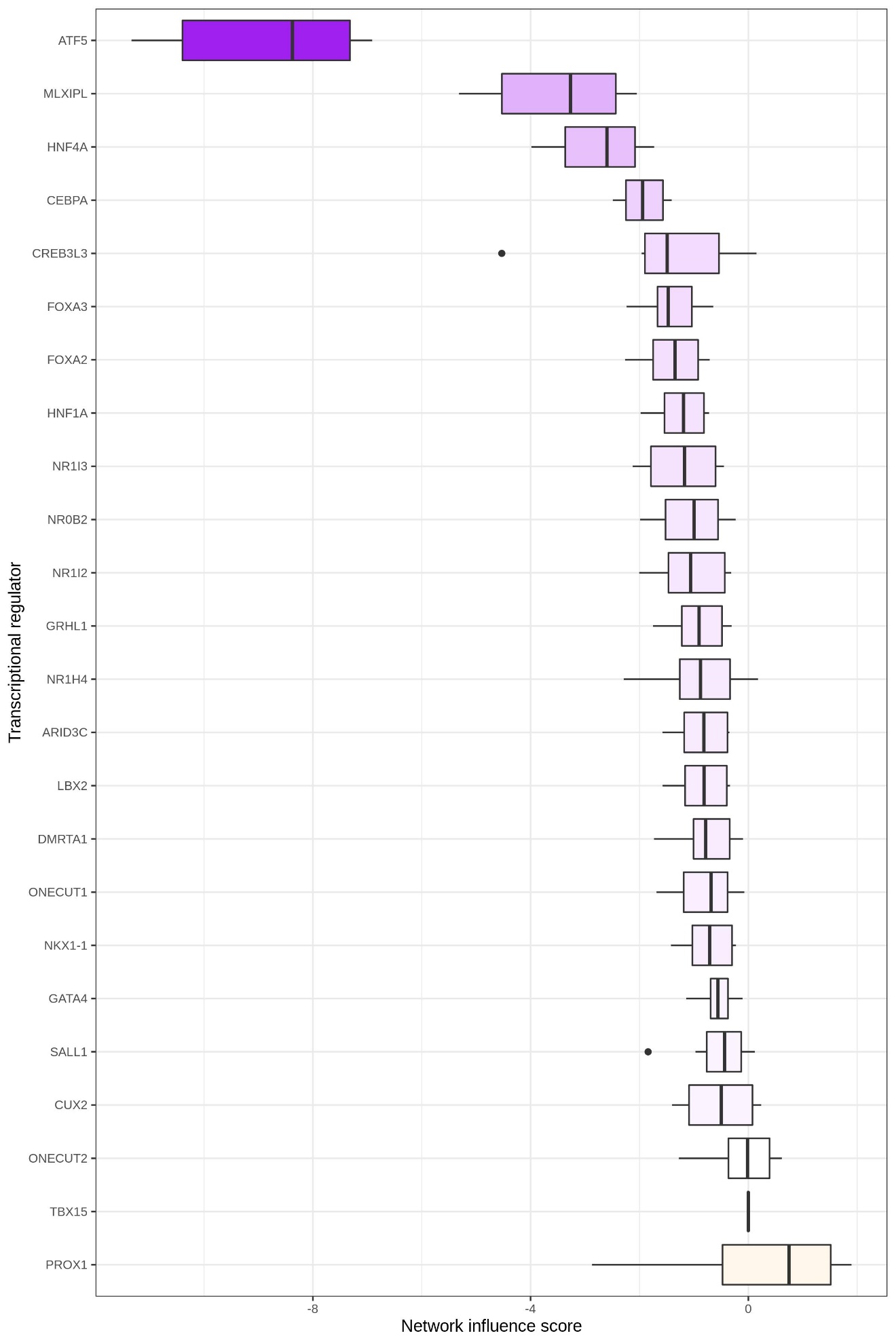


(e)
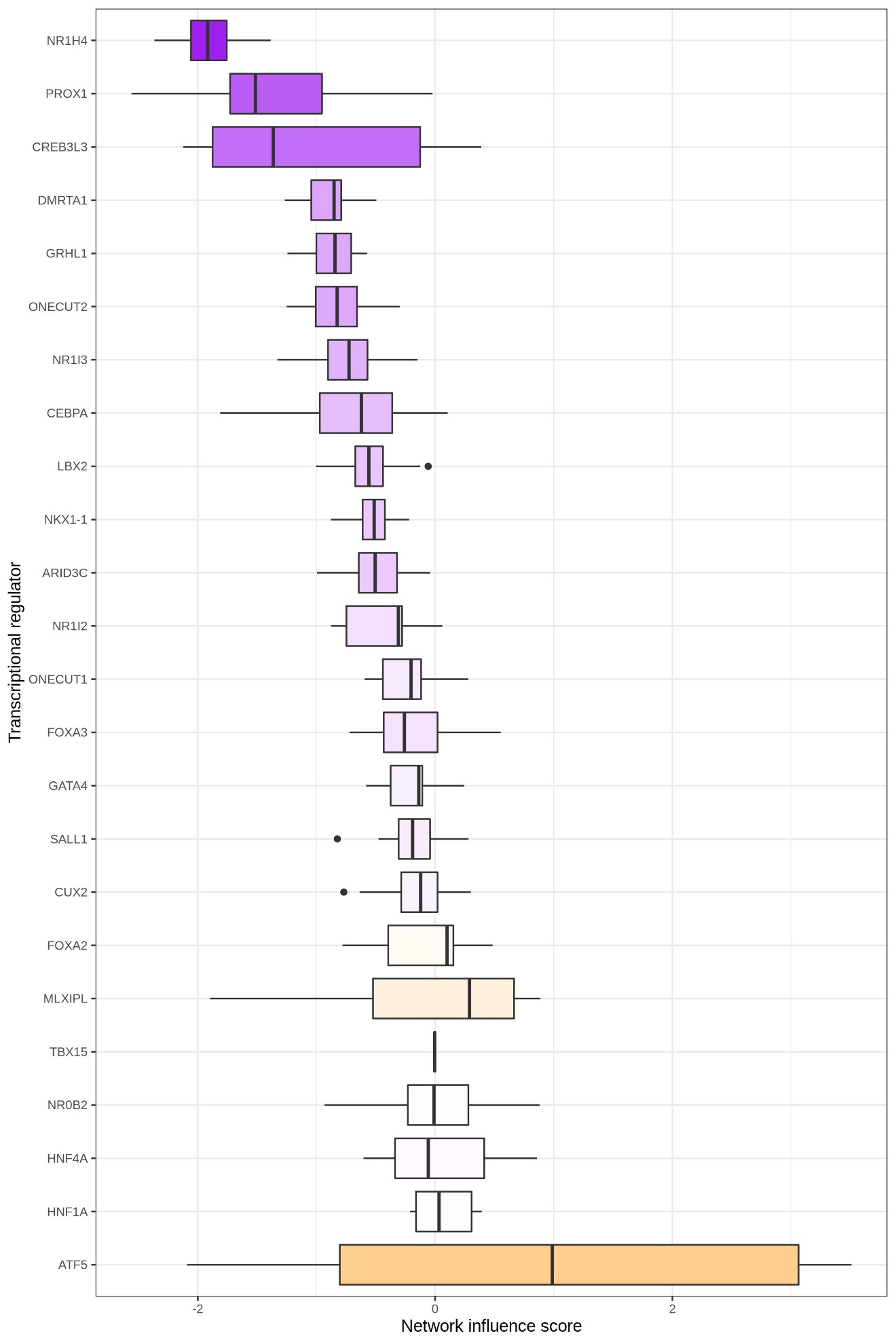


(f)
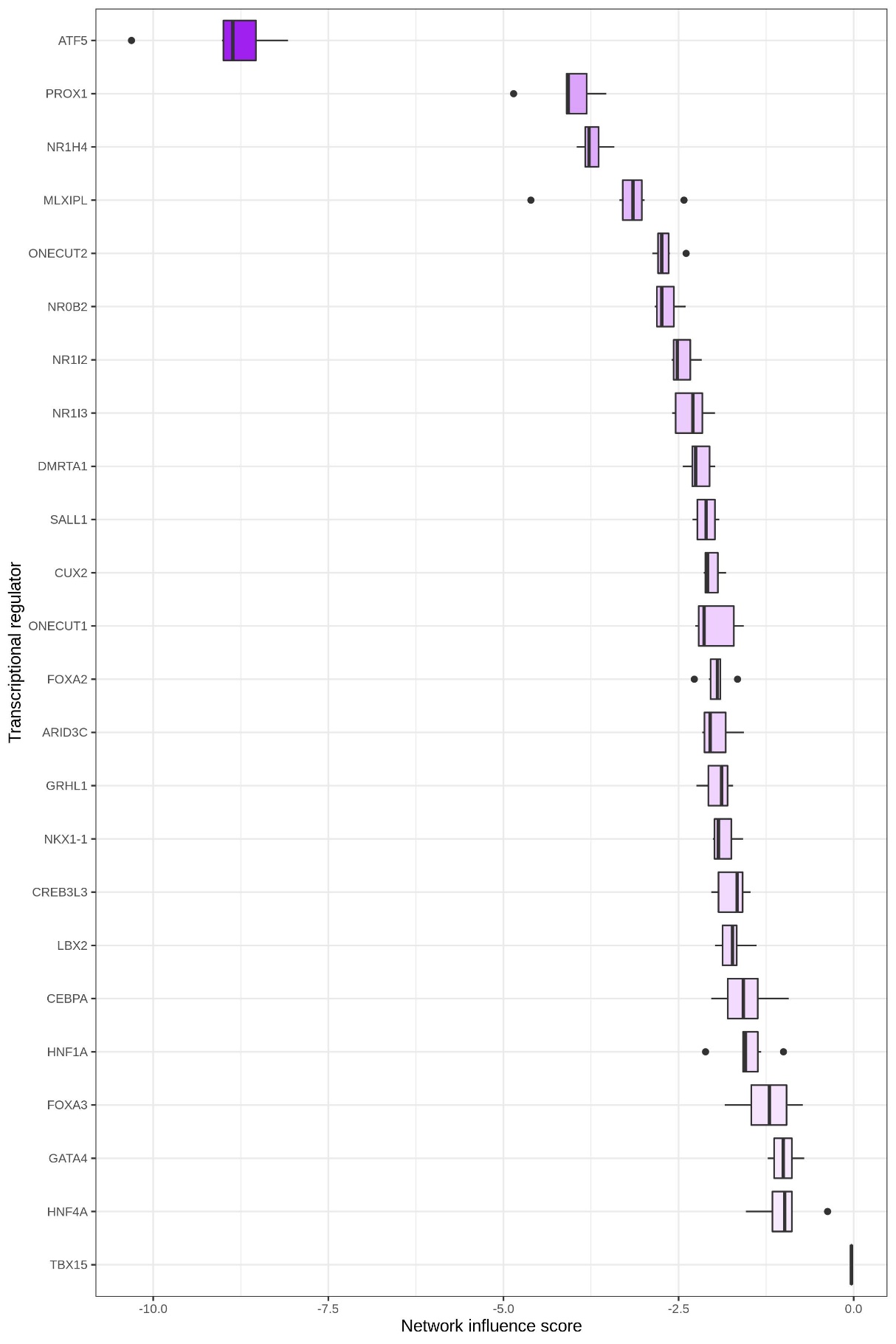


Suppl. Figure 5: NIS (Network Influence Score), calculated using R package CellNet, highlighting the perturbation in the expression of the transcription factors. (a) 3D liver microtissues, (b) HepaRG 3D, (c) HepG2, (d) hPCLiS, (e) PHH, and (f) iPSC-HLCs.

1. **Supplementary tables**

Suppl. Table 1: The samples removed after filtration.

Suppl. Table 2: Classification score for *in vitro* liver cell models predicted using CellNet.

Suppl. Table 3: List of non-DEGs for all cell models

Suppl. Table 4: Pathway mapping by (a) non-DEGs, (b) DEGs, and (c) non-DEGs^DTU-^ from all cell models on all KEGG pathways for humans. The values correspond to number of ensembl gene ids mapped to the particular pathway.

Suppl. Table 5: Cell processes and pathways taken from KEGG for comparing the different liver cell models. The process categorization is based on information from [[8](#_ENREF_8)].

| **Processes** | **Pathway** |
| --- | --- |
|  | |
| **Cell processes** | Cell cycle |
|  | Apoptosis |
|  | Primary bile acid biosynthesis |
| **Liver regrowth and regeneration** | Wnt signaling pathway |
|  | Notch signaling pathway |
|  | Hedgehog signaling pathway |
| **Cancer** | Hepatocellular carcinoma |
| **Viral infection** | Hepatitis C |
| **Immune response and inflammation** | NF-kappa B signaling pathway |
|  | T cell receptor signaling pathway |
|  | B cell receptor signaling pathway |
| **Drugs and xenobiotics metabolism** | Metabolism of xenobiotics by cytochrome P450 |
|  | Drug metabolism - cytochrome P450 |
|  | Drug metabolism - other enzymes |
| **Liver toxicity** | Non-alcoholic fatty liver disease (NAFLD) |
| **DNA repair** | Base excision repair |
|  | Nucleotide excision repair |
|  | Mismatch repair |
|  | Homologous recombination |
|  | Non-homologous end-joining |

Suppl. Table 6: List of DEGs for all cell models

Suppl. Table 7: List of non-DEG^DTU-^ for all cell models

Suppl. Table 8: List of DTU for all cell models

**References**

1. Moya M, Benet M, Guzman C, Tolosa L, Garcia-Monzon C, Pareja E, Castell JV, Jover R: **Foxa1 reduces lipid accumulation in human hepatocytes and is down-regulated in nonalcoholic fatty liver**. *PloS one* 2012, **7**(1):6.

2. Gu X, Albrecht W, Edlund K, Kappenberg F, Rahnenführer J, Leist M, Moritz W, Godoy P, Cadenas C, Marchan R: **Relevance of the incubation period in cytotoxicity testing with primary human hepatocytes**. *Archives of toxicology* 2018:1-11.

3. Vanhove J, Pistoni M, Welters M, Eggermont K, Vanslembrouck V, Helsen N, Boon R, Najimi M, Sokal E, Collas P *et al*: **H3K27me3 Does Not Orchestrate the Expression of Lineage-Specific Markers in hESC-Derived Hepatocytes In Vitro**. *Stem Cell Reports* 2016, **7**(2):192-206.

4. Helsen N, Debing Y, Paeshuyse J, Dallmeier K, Boon R, Coll M, Sancho-Bru P, Claes C, Neyts J, Verfaillie CM: **Stem cell-derived hepatocytes: A novel model for hepatitis E virus replication**. *J Hepatol* 2016, **64**(3):565-573.

5. Bell CC, Hendriks DFG, Moro SML, Ellis E, Walsh J, Renblom A, Fredriksson Puigvert L, Dankers ACA, Jacobs F, Snoeys J *et al*: **Characterization of primary human hepatocyte spheroids as a model system for drug-induced liver injury, liver function and disease**. *Sci Rep-Uk* 2016, **6**:25187-25187.

6. Granitzny A, Knebel J, Schaudien D, Braun A, Steinberg P, Dasenbrock C, Hansen T: **Maintenance of high quality rat precision cut liver slices during culture to study hepatotoxic responses: Acetaminophen as a model compound**. *Toxicology in Vitro* 2017, **42**:200-213.

7. Radley AH, Schwab RM, Tan Y, Kim J, Lo EKW, Cahan P: **Assessment of engineered cells using CellNet and RNA-seq**. *Nature Protocols* 2017, **12**:1089.

8. Dufour J-F, Clavien P-A, Graf R, Trautwein C: **Signaling pathways in liver diseases**: Springer; 2010.
