# Supplementary figures and images for: Comparing in vitro human liver models to in vivo human liver using RNA-Seq
Source: Arch Toxicol. 2020 Oct 27;95(2):573–89. doi: 10.1007/s00204-020-02937-6 (PMC7870774; doi:10.1007/s00204-020-02937-6)

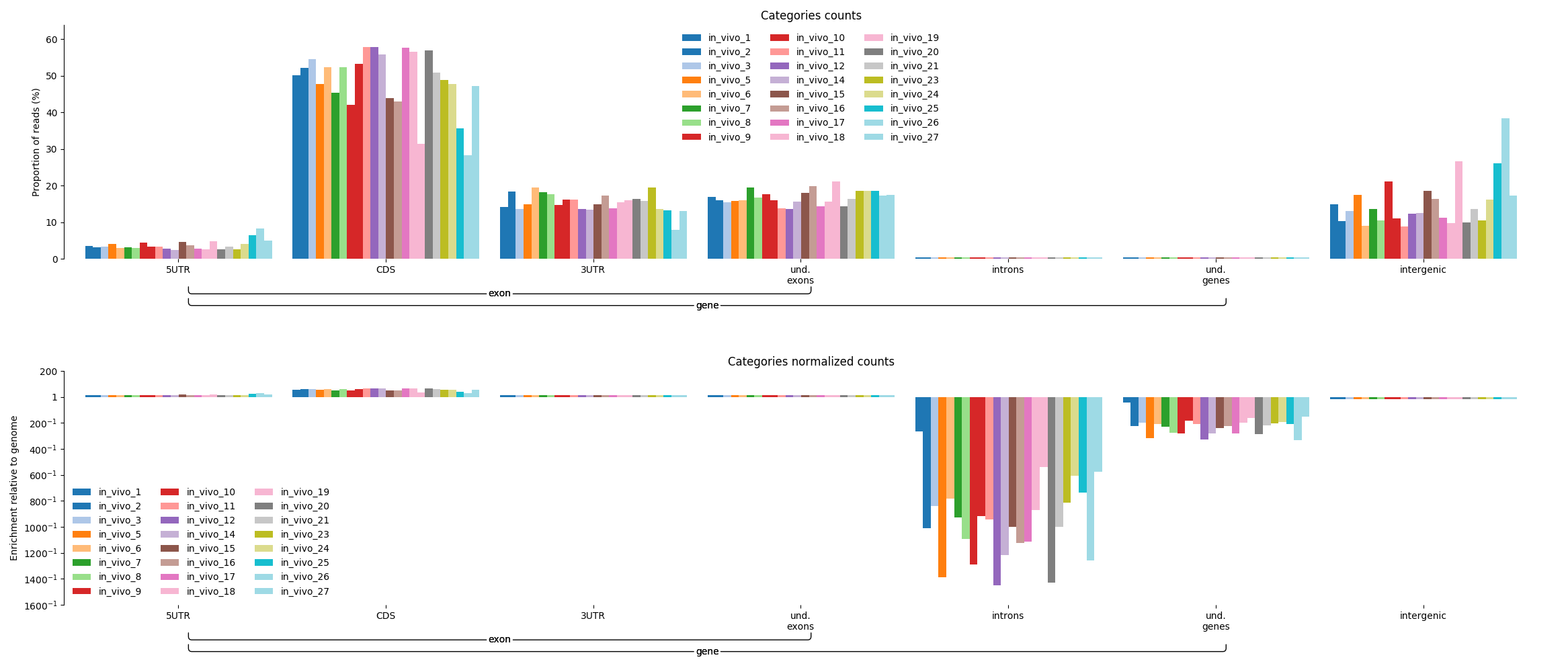

Supplement: Supplementary file 3 — Supplementary file3 (DOCX 89 kb) [file 204_2020_2937_MOESM3_ESM.png]

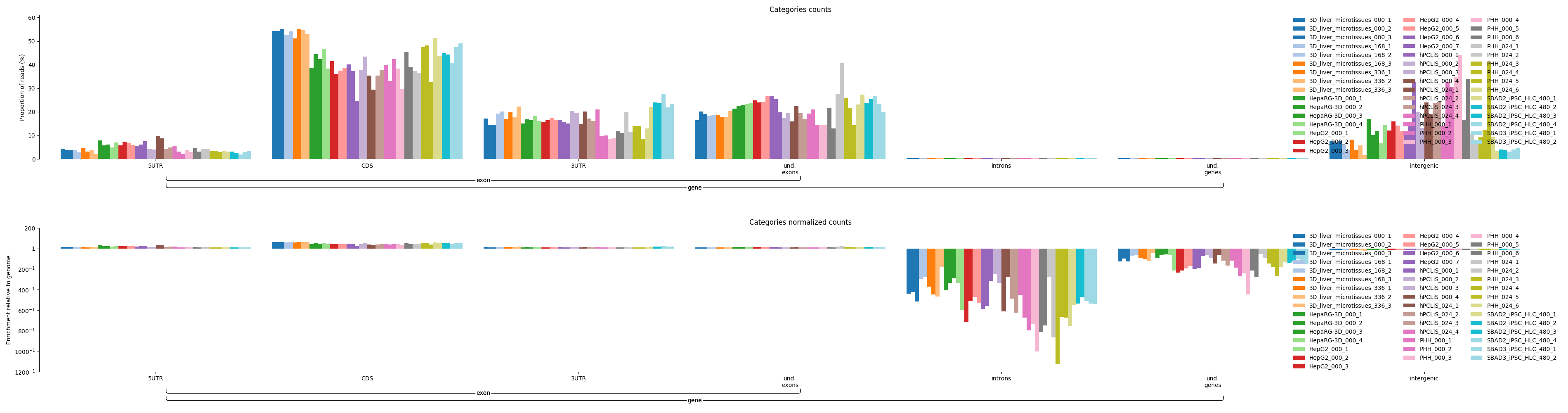

Supplement: Supplementary file 4 — Supplementary file4 (DOCX 254 kb) [file 204_2020_2937_MOESM4_ESM.png]

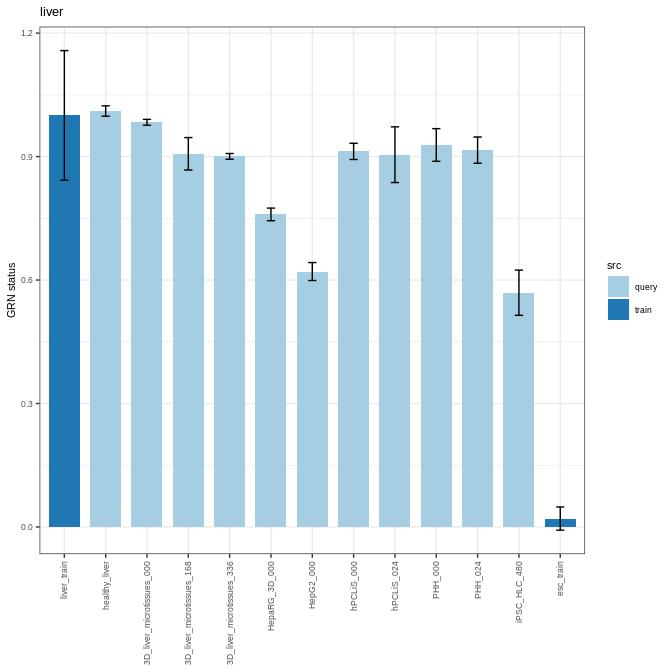

Supplement: Supplementary file 5 — Supplementary file5 (DOCX 39 kb) [file 204_2020_2937_MOESM5_ESM.jpeg]

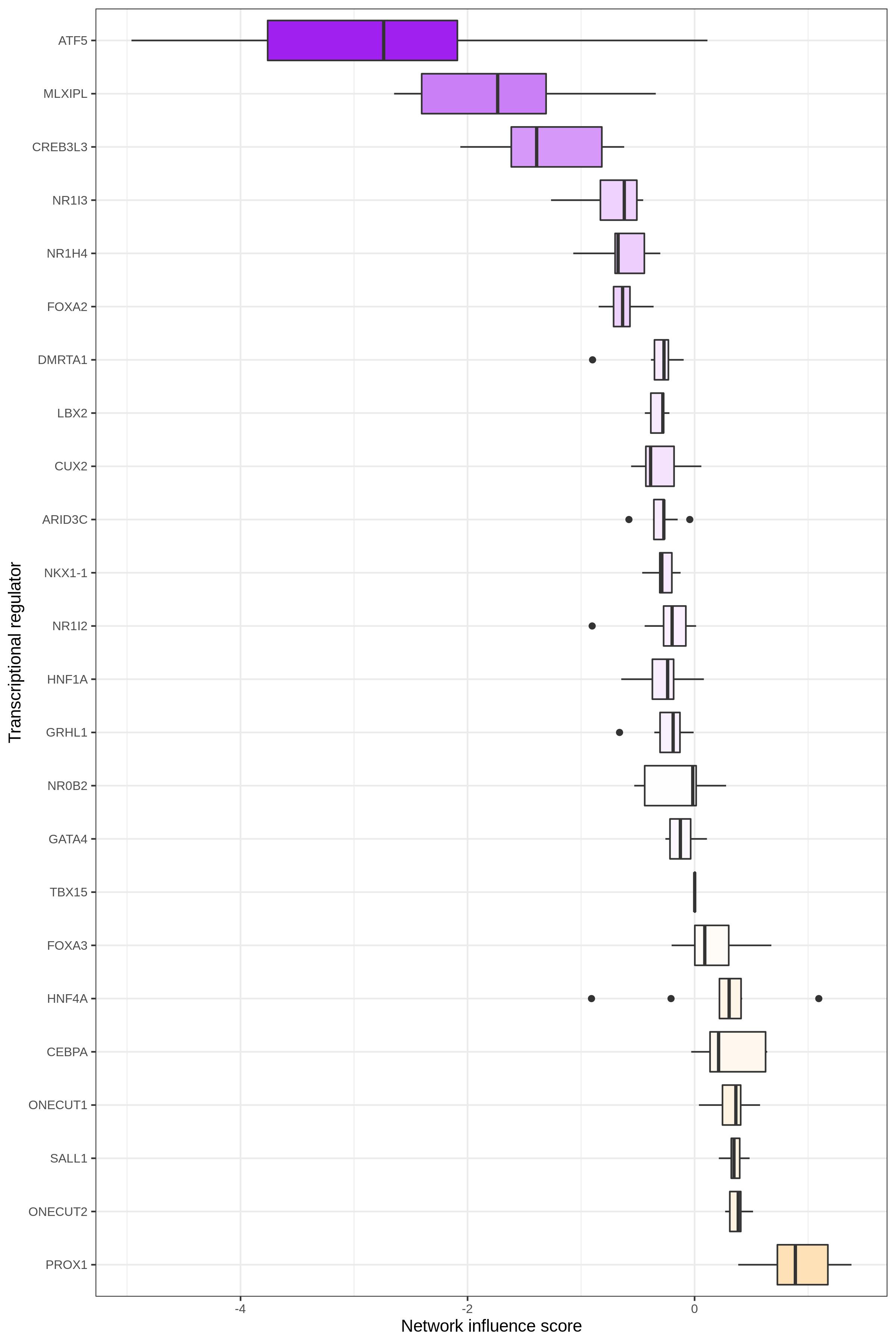

Supplement: Supplementary file 6 — Supplementary file6 (DOCX 324 kb) [file 204_2020_2937_MOESM6_ESM.jpeg]

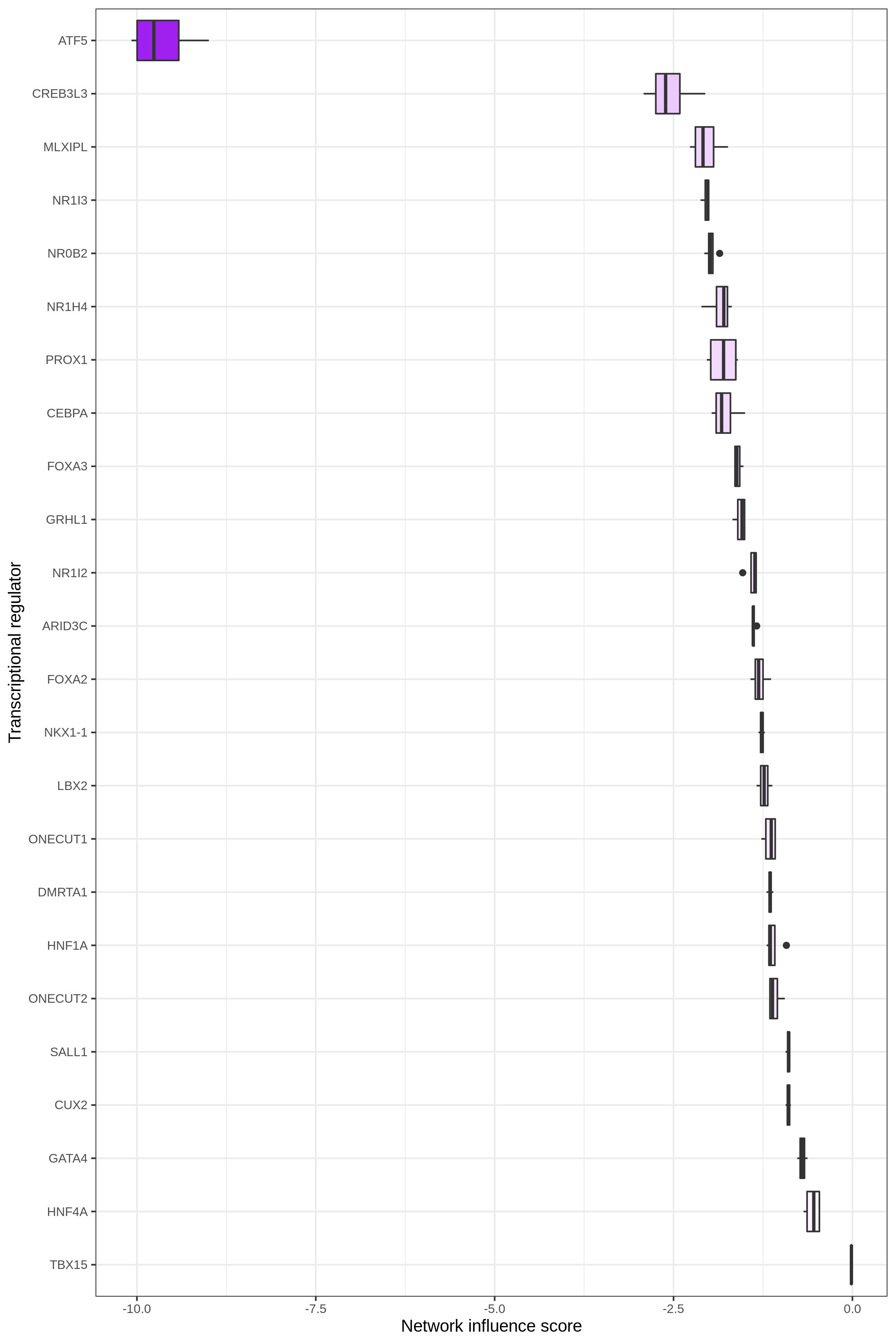

Supplement: Supplementary file 7 — Supplementary file7 (DOCX 312 kb) [file 204_2020_2937_MOESM7_ESM.jpeg]

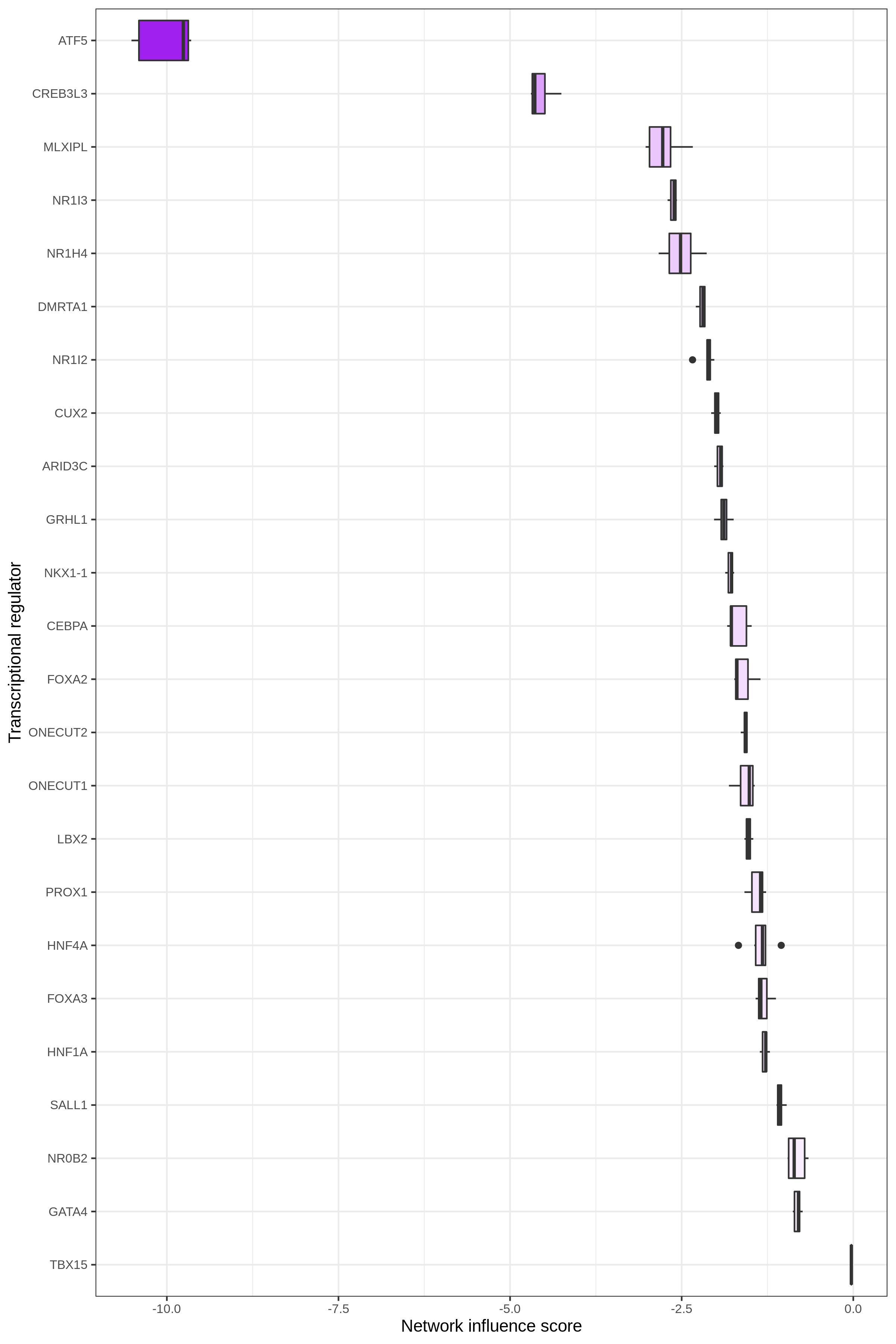

Supplement: Supplementary file 8 — Supplementary file8 (DOCX 316 kb) [file 204_2020_2937_MOESM8_ESM.jpeg]

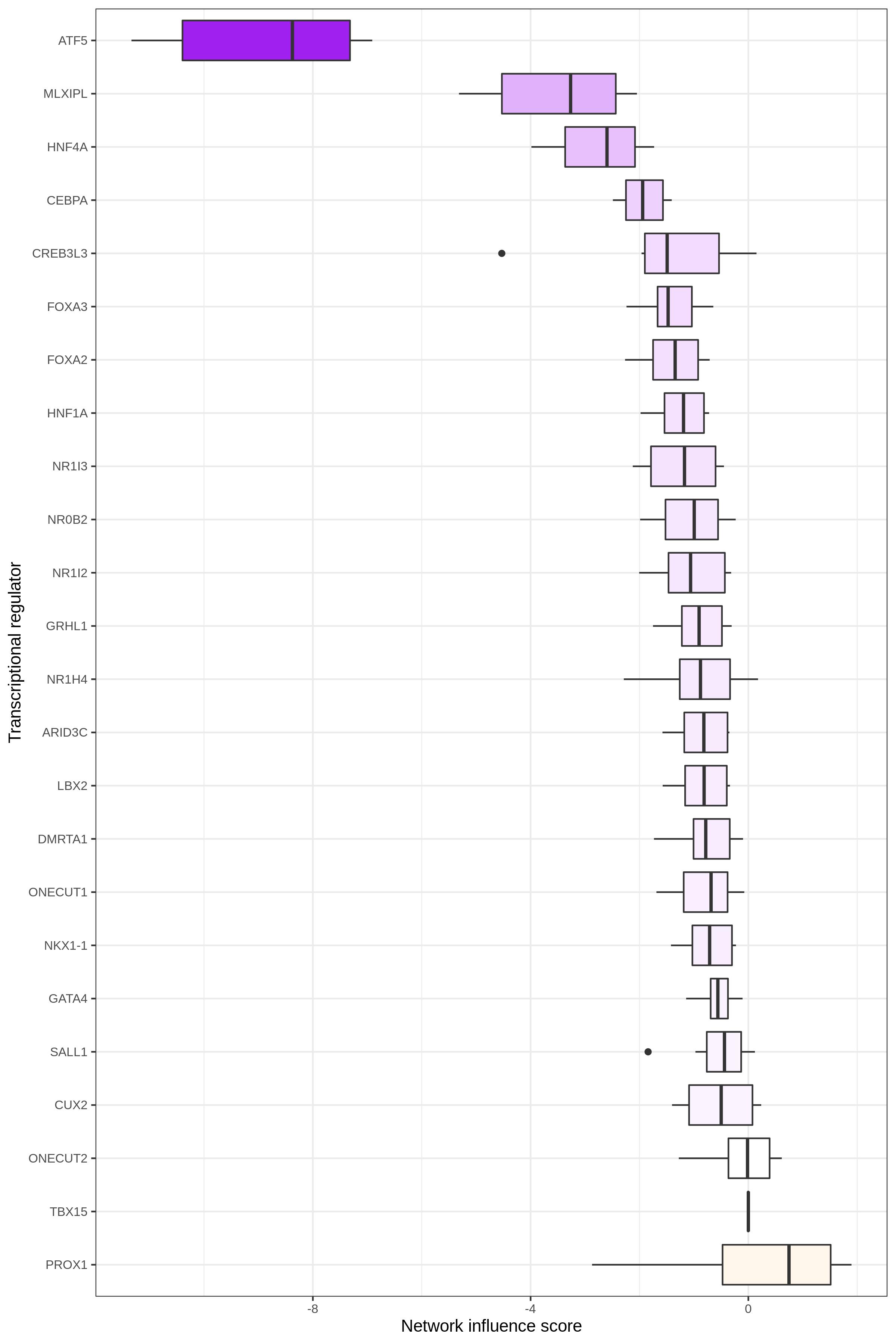

Supplement: Supplementary file 9 — Supplementary file9 (DOCX 330 kb) [file 204_2020_2937_MOESM9_ESM.jpeg]

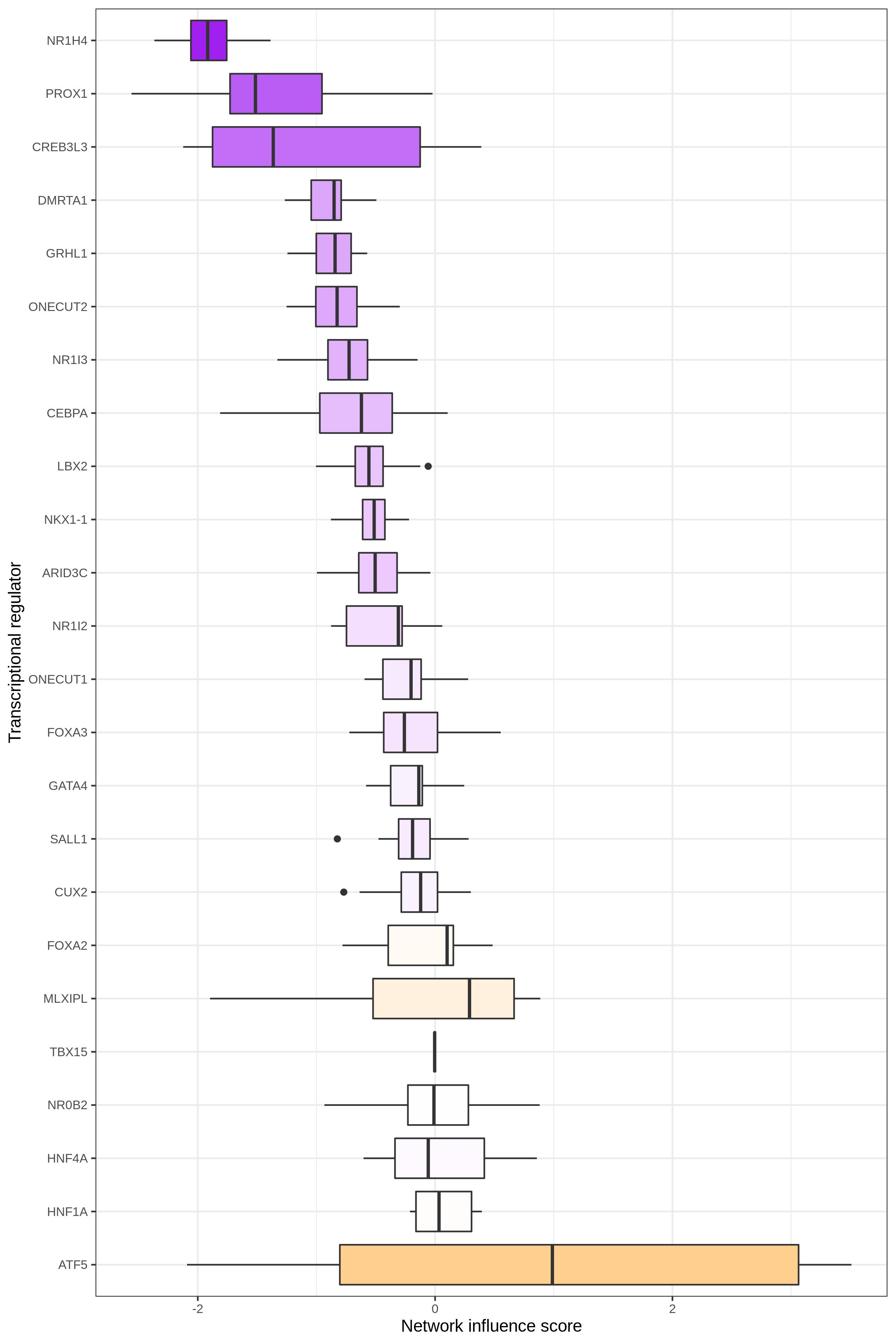

Supplement: Supplementary file 10 — Supplementary file10 (DOCX 341 kb) [file 204_2020_2937_MOESM10_ESM.jpeg]

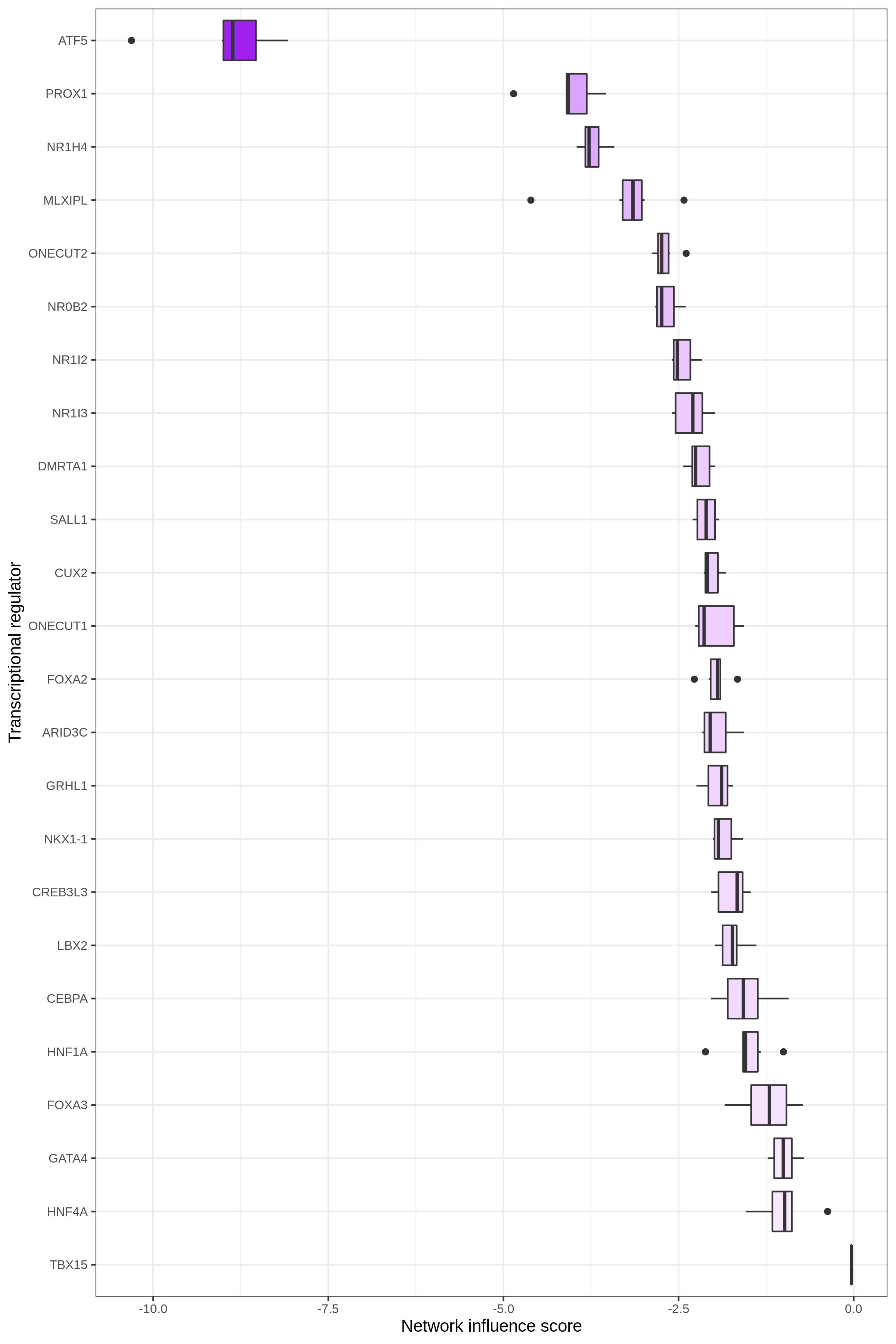

Supplement: Supplementary file 11 — Supplementary file11 (DOCX 326 kb) [file 204_2020_2937_MOESM11_ESM.jpeg]

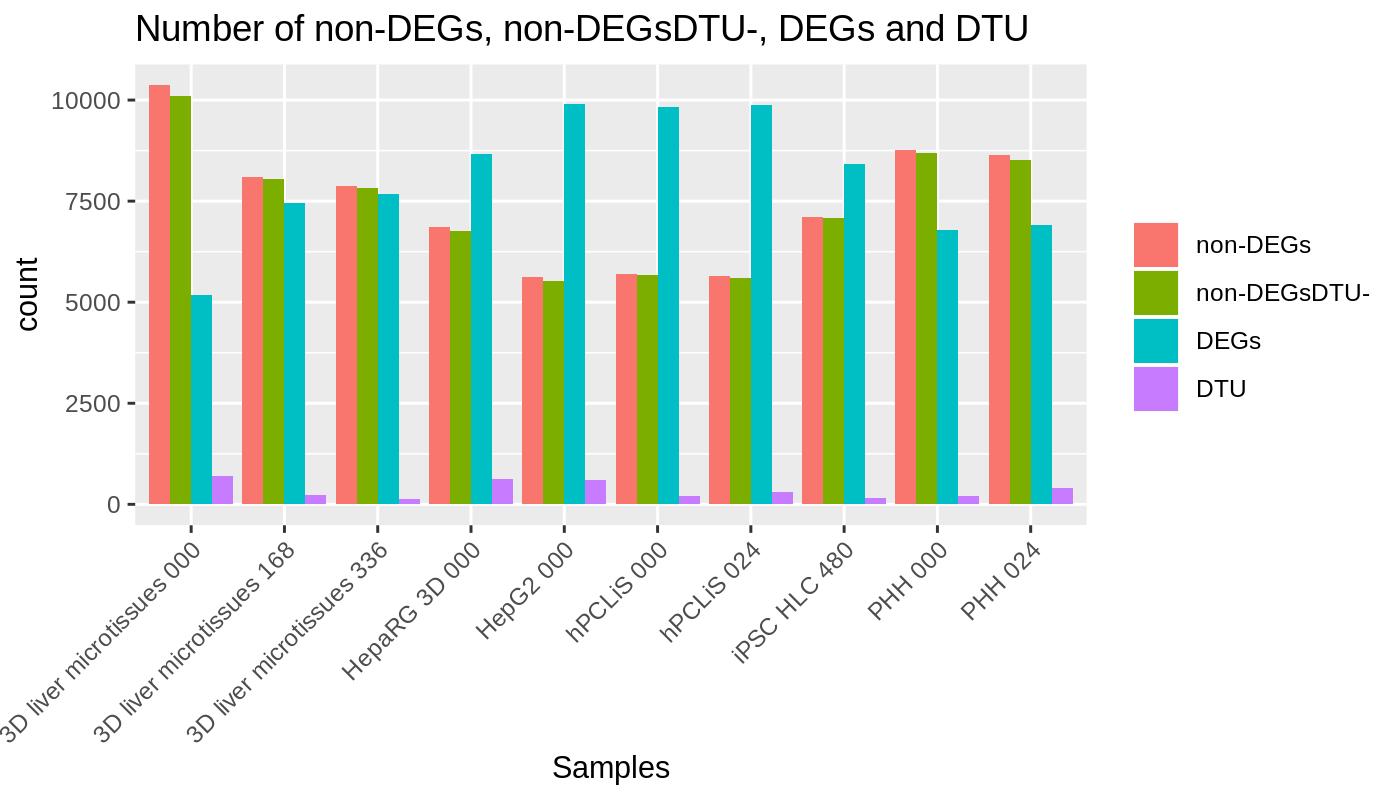

Supplement: Supplementary file 12 — Supplementary file12 (DOCX 90 kb) [file 204_2020_2937_MOESM12_ESM.jpeg]
